# Supplementary material for: Efficacy and safety of pyronaridine-artesunate (PYRAMAX) for the treatment of P. falciparum uncomplicated malaria in African pregnant women (PYRAPREG): study protocol for a phase 3, non-inferiority, randomised open-label clinical trial
Source: BMJ Open. 2023 Oct 9;13(10):e065295. doi: 10.1136/bmjopen-2022-065295 (PMC10565244; doi:10.1136/bmjopen-2022-065295)
Supplement: Supplementary data [file bmjopen-2022-065295supp001.pdf]

Table legend

Table 1: Definition of early and late treatment failures

Table 2: Study visit schedule

Table 1: Definition of early and late treatment failures

**Early treatment failure (ETF)** one of the following	**Late treatment failure (LTF)**
1) Development of danger signs or severe malaria on Days 0-3 with parasitaemia<br><br>2) Presence of parasitaemia on Day 3 with fever (axillary temperature ≥ 37.5°C)	Late Clinical Failure (LCF):<br>1) Development of danger signs or severe malaria on any day after Day 3 in the presence of parasitaemia, without having previously met any of the ETF criteria;<br>2) Presence of parasitaemia and fever on any day after Day 3, without having previously met the ETF criteria.<br><br>Late parasitological failure (LPF):<br>Presence of parasitaemia on any day, starting on Day 7, and axillary temperature <37.5 ºC, without previously meeting any of the ETF or LCF criteria.

Table 2: Study visit schedule

**Day**	**0**	**1**	**2**	**3**	**7**(± 1)	**14** (± 1)	**21** (± 2)	**28** (± 2)	**35** (± 3)	**42** (± 3)	**49-56** (± 3)	**63** (± 3)	**Any other day[1]**	**Delivery**	**4-6 weeks post-end of pregnancy**	**1-year post-end of pregnancy**
History (symptoms)	X	X	X	X	X	X	X	X	X	X	X	X	X			
Informed consent	X															
Examination (clinical)	X	X	X	X	X	X	X	X	X	X	X	X	X	X		
Vital signs	X	X	X	X	X	X	X	X	X	X	X	X	X	X		
Foetal viability	X			X	X	X	X	X	X	X	X	X	X			
Blood Pressure	X	X	X	X	X	X	X	X	X	X	X	X	X	X		
Body Temperature	X	X	X	X	X	X	X	X	X	X	X	X	X	X		
ECG	X		X		X[2]											
Blood slide[3]	X	X	X	X	X	X	X	X	X	X	X	X	X	X		
Filter paper for genotyping	X	X	X	X	X	X	X	X	X	X	X	X	X	X		
Adverse drug reactions	X	X	X	X	X	X	X	X	X	X	X	X	X	X	X	
Concomitant medications	X	X	X	X	X	X	X	X	X	X	X	X	X	X	X	
Haematology	X				X	X		X		X		X	X	X[6]		
Treatment administration	X	X	X													
Blood sample for PK[4]	X	X	X		X	X	X	X		X						
Biochemistry[5]	X	X			X											
Urine analysis	X							X		X		X				
Placenta biopsy														X		
Newborn Assessment														X[8]	X	
Newborn LFT														X[7]		
Infant assessment																X

[1]Spontaneous attendance to the health facility (unscheduled visit); [2]ECG at Day 7 if abnormal at Day 2; [3]Thick and thin blood film; [4]Four blood samples at scheduled times, first sampling before treatment administration;[5] In the event of increased LFTs >3xULN, the result will be verified by taking a further sample for analysis as soon as possible (within 24 hours of the original sample). Subsequent blood samples for LFTs will be taken at 48-hour intervals until the results return to ≤2xULN;[6] Only Hb; [7]If delivery within or 2 weeks after the active follow up; [8]Blood sample collected systematically for dried blood spots, blood slide and Hb.
