# Supplementary material for: Efficacy and safety of pyronaridine-artesunate (PYRAMAX) for the treatment of P. falciparum uncomplicated malaria in African pregnant women (PYRAPREG): study protocol for a phase 3, non-inferiority, randomised open-label clinical trial
Source: BMJ Open. 2023 Oct 9;13(10):e065295. doi: 10.1136/bmjopen-2022-065295 (PMC10565244; doi:10.1136/bmjopen-2022-065295)
Supplement: Author's manuscript [file bmjopen-2022-065295.draft_revisions.pdf]

BMJ Open is committed to open peer review. As part of this commitment we make the peer review history of every article we publish publicly available.

When an article is published we post the peer reviewers' comments and the authors' responses online. We also post the versions of the paper that were used during peer review. These are the versions that the peer review comments apply to.

The versions of the paper that follow are the versions that were submitted during the peer review process. They are not the versions of record or the final published versions. They should not be cited or distributed as the published version of this manuscript.

BMJ Open is an open access journal and the full, final, typeset and author-corrected version of record of the manuscript is available on our site with no access controls, subscription charges or pay-per-view fees (http://bmjopen.bmj.com).

If you have any questions on BMJ Open's open peer review process please email info.bmjopen@bmj.com

BMJ Open

Efficacy and safety of pyronaridine-artesunate (PYRAMAX®) for the treatment of Plasmodium falciparum malaria in African pregnant women (PYRAPREG): study protocol for a phase 3, non-inferiority, randomized open-label clinical trial

Journal:	*BMJ Open*
Manuscript ID	bmjopen-2022-065295
Article Type:	Protocol
Date Submitted by the Author:	10-Nov-2022

Complete List of Authors:	Djimde, Moussa; University of Sciences Techniques and Technologies of Bamako, Malaria Research and Training Center<br>Tshiongo, Japhet ; Universite de Kinshasa, Départment of Tropical Médecine<br>Muhindo, Hypolite ; Universite de Kinshasa Faculte de Medecine, Department of Tropical Medicine<br>Tinto, Halidou; Institut de Recherche en Sciences de la Santé (IRSS) – Unité de Recherche Clinique de Nanoro, Burkina Faso Ouagadougou, BF<br>Sevene, Esperanca; Centro de Investigacao em Saude de Manhica; Universidade Eduardo Mondlane<br>Traore, Maminata; Institut de Recherche en Sciences de la Santé (IRSS) – Unité de Recherche Clinique de Nanoro, Burkina Faso<br>Vala, Anifa; Centro de Investigação em Saúde de Manhiça<br>Macuacua, Salesio; Centro de Investigação em Saúde da Manhiça (CISM), Mozambique<br>Kabore, Berenger; Institut de Recherche en Sciences de la Santé (IRSS) – Unité de Recherche Clinique de Nanoro, Burkina Faso<br>Dabira, Edgard; MRC Unit The Gambia (MRCG) at the London School of Hygiene and Tropical Medicine, The Gambia London, UK<br>Erhart, Annette; MRC Unit The Gambia (MRCG) at the London School of Hygiene and Tropical Medicine, The Gambia<br>Diakite, Hamadoun ; University of Sciences Techniques and Technologies of Bamako, Malaria Research & Training Centre<br>Keita, Mohamed; University of Sciences Techniques and Technologies of Bamako Faculty of Medicine Odontostomatology<br>Piqueras, Mireia; Instituto de Salud Global Barcelona Barcelona, Catalunya, ES<br>González, Raquel; Instituto de Salud Global Barcelona Barcelona, Catalunya, ES<br>Menendez, Clara; Instituto de Salud Global Barcelona Barcelona, Catalunya, ES Barcelona, ES<br>Dorlo, Thomas; Netherlands Cancer Institute / Antoni van Leeuwenhoek Hospital, Amsterdam, the Netherlands<br>Sagara, Issaka; Universite des Sciences des Techniques et des Technologies de Bamako, Malaria Research and Training Center (MRTC)<br>Mens, Petra; Amsterdam University Medical Centres, Academic Medical Centre at the University of Amsterdam (AMC), Amsterdam, Netherlands<br>Schallig, Henk; Amsterdam University Medical Centres, Academic Medical

	Centre at the University of Amsterdam (AMC), Amsterdam, Netherlands<br>D'Alessandro, Umberto; MRC Laboratories The Gambia<br>Kayentao, Kassoum; University of Sciences Techniques and Technologies of Bamako, Malaria Research & Training Centre
Keywords:	EPIDEMIOLOGY, Infection control < INFECTIOUS DISEASES, Tropical medicine < INFECTIOUS DISEASES, PARASITOLOGY

Paper draft Version 10
November 6th , 2022

Administrative information

Efficacy and safety of pyronaridine-artesunate (PYRAMAX®) for the treatment of *P. falciparum* malaria in African pregnant women (PYRAPREG): study protocol for a phase 3, non-inferiority, randomized open-label clinical trial

Moussa Djimde[1,2¥], Japhet Kabalu Tshiongo[2,3¥], Hypolite Muhindo Mavoko[3], Halidou Tinto[4], Esperanca Sevene[5,6], Maminata Traoré[4], Anifa Vala[5], Salesio Macuacua[5], Bérenger Kaboré[4], Edgard Diniba Dabira[7], Annette Erhart[7], Hamadoun Diakité[1], Mohamed Keita[1], Mireia Piqueras[8], Raquel González[5,8], Clara Menéndez[5,8], Thomas P.C. Dorlo[9], Issaka Sagara[1], Petra F. Mens[2], Henk D. F. H. Schallig[2], Umberto D'Alessandro[7] and Kassoum Kayentao[1*]

[¥] These authors contributed equally

Author's Affiliations:

[1]Malaria Research and Training Center (MRTC), University of Sciences of Techniques and Technologies of Bamako (USTTB), Mali

[2]Amsterdam University Medical Centres, Academic Medical Centre at the University of Amsterdam (AMC), Amsterdam, Netherlands

[3]Department of Tropical Medicine, University of Kinshasa (UNIKIN), Kinshasa, Democratic Republic of the Congo

[4]Institut de Recherche en Sciences de la Santé (IRSS) – Unité de Recherche Clinique de Nanoro, Burkina Faso

[5]Centro de Investigação em Saúde da Manhiça (CISM), Mozambique

[6]Faculty of Medicine, Eduardo Mondlane University, Mozambique

[7]MRC Unit The Gambia (MRCG) at the London School of Hygiene and Tropical Medicine, The Gambia

[8]Barcelona Institute for Global Health (ISGlobal), Hospital Clínic-Universitat de Barcelona, Spain

[9] Netherlands Cancer Institute / Antoni van Leeuwenhoek Hospital, Amsterdam, the Netherlands

For peer review only - http://bmjopen.bmj.com/site/about/guidelines.xhtml

Paper draft Version 10
November 6th , 2022

Trial registration:

PACTR202011812241529

Protocol version: 7.0 12 July 2022

Trial Sponsor and role

The trial sponsor is University of Sciences, Techniques, and Technologies of Bamako (USTTB). Hamdallaye ACI 2000, Rue 405 - Porte 359, Bamako

Téléphone : +223 20 29 04 07

http://usttb.edu.ml/

The Trial sponsor in the study has the responsibility to ensure that data and source documents granted for trial monitoring, audits, DSMB and Ethics Committee review and regulatory inspections as appropriate.

***Corresponding Author**: Kassoum Kayentao (kayentao@icermali.org)

Malaria Research and Training Center (MRTC), University of Sciences of Techniques and Technologies of Bamako (USTTB), Mali

For peer review only

For peer review only - http://bmjopen.bmj.com/site/about/guidelines.xhtml

Paper draft Version 10
November 6th , 2022

Abstract

Introduction: Malaria infection during pregnancy increases the risk of low birthweight and infant mortality and should be prevented and treated. Artemisinin-based combination treatments are generally well tolerated, safe and effective, the most used being artemether-lumefantrine (AL) and dihydroartemisinin-piperaquine (DP). Pyronaridine-artesunate (PA) is a new artemisinin-based combination. The main objective of this study is to determine the efficacy, safety, and tolerability of PA versus AL or DP when administered to pregnant women with confirmed *P. falciparum* infection in the second or third trimester. The primary hypothesis is the pair-wise non-inferiority of PA as compared to either AL or DP.

Methods and analysis: A phase 3, non-inferiority, randomized, open-label clinical trial to determine the safety and efficacy of AL, DP, and PA in pregnant women with malaria in 5 sub-Saharan, malaria-endemic countries (Burkina Faso, Democratic Republic of the Congo, Mali, Mozambique and The Gambia). A total of 1,875 pregnant women will be randomized to one of the treatment arms. Women will be actively monitored until the Day 63 post-treatment, at delivery and 4-6 weeks after delivery, and infants' health will be checked at their first birthday. The primary endpoint is the PCR-adjusted rate of adequate clinical and parasitological response at Day 42 in the per protocol population.

Ethics and Dissemination: This protocol has been approved by Ethics Committee for Health Research in Burkina Faso, the National Health Ethics Committee in Democratic Republic of Congo, the Ethics Committee of the Faculty of Medicine and Odontostomatology / Faculty of Pharmacy in Mali in Mali, The Gambia Government/MRCG Joint Ethics Committee and the National Bioethics Committee for Health in Mozambique. Written informed consent will be taken from each individual prior to her participation in the study. The results will be published in peer reviewed Open Access Journals and presented at (inter)national conferences and meetings.

Keywords:

Malaria, pyronaridine-artesunate, artemether-lumefantrine, dihydroartemisinin-piperaquine, pregnant women, Burkina Faso, Democratic Republic of the Congo, Mali, Mozambique, The Gambia.

For peer review only - http://bmjopen.bmj.com/site/about/guidelines.xhtml

Strengths and limitations of this study

- This is randomized clinical trial that will provide evidence on the safety and efficacy of a newly registered Artemisinin-Based Combination (Pyronaridine-Artesunate (PA)) for the treatment of uncomplicated malaria in African pregnant women during the second and third trimester of pregnancy.
- The trial will generate additional data on the safety and efficacy of dihydroartemisinin-piperaquine, and artemether-lumefantrine and will increase substantially the treatment options for malaria in pregnancy.
- As PA is the last Artemisinin-Based Combination to have been registered for the treatment of malaria, the proposed trial will complement the information already available for malaria treatment in children and non-pregnant adults.
- Weaknesses include the lack of regular monitoring of children within the first year of birth and the non-inclusion of infected women in the first trimester.

For peer review only

For peer review only - http://bmjopen.bmj.com/site/about/guidelines.xhtml

Paper draft Version 10
November 6th , 2022

INTRODUCTION

The risk of malaria infection is higher during pregnancy due to the related immunological and hormonal changes (1). Malaria during pregnancy increases the risk of low birth weight (LBW) which is associated with higher infant mortality (2). The prevention and control of malaria during pregnancy is based on insecticide-treated nets (ITNs), intermittent preventive treatment during pregnancy (IPTp) with sulfadoxine-pyrimethamine (SP), and effective case management of clinical malaria, including anaemia (3). The latter relies on artemisinin-based combination therapies (ACTs) which are well tolerated, safe and efficacious (4). Their use is currently limited to the second and third trimester although the risk of miscarriage, stillbirths or major birth defects when used during the first trimester is better to quinine, the recommended drug (5,6).

Currently available ACTs for the treatment of malaria in pregnancy, namely artemether-lumefantrine (AL), artesunate-amodiaquine (ASAQ), mefloquine-artesunate (MAS) and dihydroartemisinin-piperaquine (DP), are generally safe and efficacious (4). Nevertheless, when considering efficacy and tolerability, DP is probably the best option as it provides a long post-treatment prophylaxis and is well tolerated. DP is also considered for IPTp, as a possible alternative to SP where resistance to this treatment is high (7). Its deployment as IPTp would significantly limit its use as curative treatment. There is the need of increasing the therapeutic options for treating malaria during the 2nd and 3rd trimesters of pregnancy. Pyronaridine-artesunate (PA) may be a possible candidate. PA tablets and granules (Pyramax®) have received a positive European Medicines Agency /Article 58 scientific opinion for the treatment of acute uncomplicated *P. falciparum* or *P. vivax* malaria (8). The treatment (tablets) is currently registered in 19 African countries and 5 Asian countries. Pyramax (granules) is registered in 15 African countries.

To address this need, we designed a randomized open-label clinical trial to determine the efficacy, safety, and tolerability of PA versus AL or DP when administered to pregnant women with confirmed *P. falciparum* infection in the second or third trimester.

The specific trial objectives are:

A. To compare the efficacy of PA versus AL or DP in terms of: (i) treatment success (see definition below) 28, 42 and 63 days after the start of treatment, with and without genotyping; (ii) parasite clearance time, including sub-microscopic malaria infections; (iii) gametocyte carriage and clearance; (iv) haematological recovery by Day 7, 14,

For peer review only - http://bmjopen.bmj.com/site/about/guidelines.xhtml

28, 42 and 63 after treatment and at delivery; (v) Birth weight measured within 72 hours of delivery and (vi) placental *P. falciparum* malaria.

B. To describe the safety profile of PA, AL and DP in terms of: (i) tolerability and (ii) adverse events (AEs), including serious adverse events (SAEs) and adverse events of special interest (AESI), during the 63-day post-treatment follow-up (mother), at delivery (mother and baby), one month (mother and baby) and one year after the end of pregnancy (baby).

C. To explore the pharmacokinetics of pyronaridine in HIV-infected and non-HIV-infected pregnant women. This will be done on a small number of study subjects (60 women, 30 HIV uninfected and 30 HIV infected), in the 2 countries with the highest HIV prevalence, namely Democratic Republic of the Congo (DRC) and Mozambique.

METHODS AND ANALYSIS

Study setting

The trial will be implemented in five African countries with multiple sites of recruitment as indicated in table 1.

Table 1 Countries and Study site characteristics

Burkina-Faso: Nanoro	The study site is situated in central-west of the country, 90 Km from Ouagadougou in the Nanoro health district catchment area. Malaria transmission is high and extremely seasonal.
Democratic Republic of the Congo: Lisungi	The trial will be carried out at Lisungi health centre, located in Kinshasa suburb, where malaria transmission is perennial.
Mali: San and Téné	San health centre is located at 440 km of northeast of Bamako and serves a population of about 160,000 inhabitants.<br>Téné is at about 50 km south of San and has a population of about 25,000 inhabitants. Malaria transmission is more intense during the rainy season.
Mozambique: Manhiça District	Manhiça is located in southern Mozambique, 80 Km north of Maputo city. Malaria is endemic with perennial transmission, mainly by *P. falciparum,* which represents one of the top five causes of mortality in under-five.

For peer review only - http://bmjopen.bmj.com/site/about/guidelines.xhtml

The Gambia: Basse, Demba Kunta Koto, Fatoto, Gambissara and Koina	The trial will be carried out in the Upper River Region (eastern part of the country), where the MRCG has a field station. There are 5 health facilities (Basse, Demba Kunta Koto, Fatoto, Gambissara and Koina) from which pregnant women can be recruited. Malaria transmission is seasonal.

Patient and public involvement

Although patients were not involved in the design of the study, this project was designed to respond to their main concern which is the reduction of malaria adverse effect in pregnant population. The government of the five African countries were engaged through their National Malaria Control Programme which objective is to provide the evidence base to make an additional ACT available for malaria treatment in pregnancy. In case of a positive result, it will be important to integrate this new treatment in (inter) national guidelines. This will be obtained by disseminating as rapidly and efficiently as possible the trial's results to relevant stakeholders, e.g. WHO, National Malaria Control Programmes (NMCPs), and the scientific community working on malaria treatment. Through meetings in all the involved countries, permission from community, religious leaders, and women representatives has been sought before the trial start. Therefore, similar channels will be used to share the study results. Participation in our trial can be burdensome as participants may endure psychological, cost, and physical impacts. However, the project provided special attention such as closer supervision and treatment of any illness to enrolled patients. Also, transport cost has been provided to participants living far from the recruitment centre. Nonetheless, it has been suggested to all participants to withdraw their consent any time if they fell burdened by the study procedures.

Study design

This is an open-label, multicentre, randomized, non-inferiority clinical trial comparing PA with AL and DP for the treatment of *P. falciparum* malaria in women in the second and third trimesters of pregnancy.

The primary hypothesis is the pair-wise non-inferiority of PA as compared to either AL or DP, defined as a difference in the Day 42 PCR-adjusted adequate clinical and parasitological response (ACPR) of <5% (non-inferiority margin).

For peer review only - http://bmjopen.bmj.com/site/about/guidelines.xhtml

Paper draft Version 10
November 6th , 2022

Participants and procedures

To be included, patients should satisfy all the inclusion criteria while none of the exclusion criteria should be present.

Inclusion criteria

1. Gestation ≥16 weeks and <37 weeks as assessed by ultrasound when possible. If not, height of the uterus or delay of menstruation will be used.
2. *P. falciparum* mono-infection (by microscopy) of any density, regardless of symptoms and HIV status.
3. Haemoglobin ≥7g/dL.
4. Age ≥15 years.
5. Residence within the health facility catchment area.
6. Willingness to adhere to study requirements and to deliver at the health facility.
7. Ability to provide written informed consent; if the woman is minor of age/not emancipated, the consent must be given by a parent or legal guardian according to national law (however, in this case, the investigator is responsible to check that the woman herself is also freely willing to take part in the study).
8. For the PK study, HIV-infected women should be on first line anti-retroviral treatment for at least 6 months.

Exclusion criteria

1. Known allergy to the study drugs.
2. History of known pregnancy complications or poor obstetric history such as repeated stillbirths or eclampsia.
3. History or presence of major illnesses likely to influence pregnancy outcome.
4. Any significant illness at the time of screening requiring hospitalization, including:
   i. Severe malaria;
   ii. Any sign or symptom suggesting hepatic lesions (e.g. nausea with abdominal pain and icterus) or severe liver disease classified as B or C by the Child-Pugh score;
   iii. Known history or evidence of clinically significant cardiovascular disorders or family history of long QT syndrome.
5. Intent to move out of the study catchment area before delivery or delivery out of the catchment area.
6. Prior enrolment in the study.

For peer review only - http://bmjopen.bmj.com/site/about/guidelines.xhtml

7. Clear evidence of recent (1 week) treatment with antimicrobials with antimalarial activity (azithromycin, clindamycin, tetracycline, quinolones, cotrimoxazole and SP). For HIV-infected pregnant women to be included in the PK sub-study, cotrimoxazole use is not an exclusion criterion.
8. Twin/multiple pregnancy.
9. Known history of G6PD deficiency or sickle cell disease.

Randomisation

Sequence generation

Women recruited in the trial will be randomly allocated to one of the three treatment arms of the study according to a randomization list generated using R software prior any study activity.

The block randomisation technique will be used to achieve balance in the allocation of participants to treatment arms. A varying size of the blocks will be setup to reduce selection bias by using random block sizes and keeping the investigator blind to the size of each block, particularly in this open randomized control arm study. The randomisation will be done per study site within each country and according to the specific sample size allocated to each site. The data management team in each country, in coordination with the study statistician and the study Data management Centre, will print the randomization code containing the study arm and put into a sealed envelope numbered sequentially and containing the treatment arm to which the patient should be allocated.

Recruitment

Women in the 2nd or 3rd trimester of pregnancy will be screened for malaria with a Rapid Diagnostic Test; positive results will be confirmed by microscopy. Women with a confirmed *P. falciparum* infection will be asked to provide a written informed consent covering all trial procedures and be assigned a screening number. All women meeting the entry criteria will be given a randomization number. Data of screened and randomized women will be kept in a logbook.

Investigational product and comparators

<u>Pyronaridine-artesunate: Pyramax®</u> (Shin Poong Pharmaceutical Company, Korea)

PA, is a film-coated tablet containing 180 mg pyronaridine tetraphosphate and 60 mg artesunate. The tablets should be taken orally once daily for three days and according to body

For peer review only - http://bmjopen.bmj.com/site/about/guidelines.xhtml

weight as follows 24-<45Kg 2 tablets; 45-<65Kg 3 tablets; ≥65Kg 4 tablets. This dosage regimen provides a daily dose of 7.2-13.8 mg/kg pyronaridine and 2.4-4.6 mg/kg artesunate. PA can be administered at any time, regardless of food consumption.

Dihydroartemisinin-piperaquine (Alfasigma, Italy)

DP is a white film-coated tablet composed of 40 mg dihydroartemisinin and 320 mg piperaquine. DP tablets should be taken orally once daily for three days as follows 24-<36Kg 2 tablets; 36-<75Kg 3 tablets; ≥75Kg 4 tablets (10) (11).

Artemether-lumefantrine: Coartem® (Novartis)

AL tablets contain 80 mg artemether and 480 mg lumefantrine. This is a fixed-dose combination of artemether (a semi synthetic artemisinin derivative) and lumefantrine (a slowly eliminated drug also referred to as benflumetol) (12). AL 80 mg/480 mg tablets should be taken orally twice daily for three days as follows to patients weighing 35 kg and above:

- 1st dose, at the time of initial diagnosis (Day 0): 1 tablet
- 2nd dose, at 8 hours after the 1st dose: 1 tablet
- 3rd dose, in the morning of the Day 1: 1 tablet
- 4th dose, in the evening of Day 1: 1 tablet
- 5th dose, in the morning of Day 2: 1 tablet
- 6th dose, in the evening of Day 2: 1 tablet

Explanation for the choice of comparators.

Pregnant women (2nd or 3rd trimester) will be recruited for the study and allocated to one of the three treatment groups. The choice of two control arms (AL and DP) is justified by AL being the most commonly used treatment for malaria in African pregnant women, while DP has several advantages compared to AL, namely higher efficacy and longer post-treatment prophylaxis.

Study outcomes

Primary endpoint

The primary endpoint is treatment efficacy determined as the proportion of women with PCR-adjusted ACPR at Day 42, i.e. all women not having met the criteria of treatment failure (Table 2). Recurrent infections classified by genotyping as new infection will not be considered as treatment failure.

For peer review only - http://bmjopen.bmj.com/site/about/guidelines.xhtml

Secondary endpoints

Safety is the main secondary endpoint and includes adverse events detected during active follow-up (63 days post-treatment), including significant changes in relevant laboratory values, those detected at delivery, at 4-6 weeks and 1 year after birth.

Others secondary endpoints include:

1. PCR-adjusted ACPR at Days 28 and 63;
2. PCR unadjusted ACPR on Days 28, 42 and 63;
3. Parasite and fever elimination time;
4. Gametocyte carriage and clearance;
5. Hematologic recovery, i.e., Hb changes between Day 0 and Days 7, 14, 28, 42, 63 and at delivery;
6. Placenta malaria (recent, past, and chronic infection);
7. Average BW and prevalence of low birth weight (<2,500g).

Exploratory endpoints

We will explore: (i) the drug exposure and key pharmacokinetic parameters of pyronaridine in HIV uninfected pregnant women and (ii) the drug exposure and key pharmacokinetic parameters of HIV-infected pregnant women on antiretroviral therapy. Drug exposure is defined as the area under the whole blood concentration versus time curve from zero to infinity, $AUC_{0-\infty}$. The main pharmacokinetic parameters that will be evaluated are absorption rate (k_a), drug clearance (CL/F) and volume of distribution (V_d/F).

Sample size

The sample size was estimated assuming an efficacy for PA (PCR adjusted at Day 42) of at least 95% and a non-inferiority margin of 5%; non-inferiority will be tested using raw pooling of country data, using a Wilson's interval of proportion difference. The lower limit of the Wilson score interval of 97.5% of the AL (or DP) proportion of ACPR - the PA proportion of ACPR must be greater than -5% to claim non-inferiority. In addition, an adjusted non-inferiority analysis will allow for country effect (15). Assuming 20% of loss to follow-up, the total sample size for 90% power would be 3*500/0.8 = 1875, or 375 pregnant women per country and 125 per arm per country. The sample size calculated will maintain the power of the adjusted stratified non-inferiority test.

Study implementation and timeline

For peer review only - http://bmjopen.bmj.com/site/about/guidelines.xhtml

Participants' assignment into study arm is under the responsibility of study qualified physicians. All physician participating in PYRAPREG study have been trained in Good Clinical Practices and all study requirements.

Pregnant women fulfilling the inclusion/exclusion criteria will be recruited during antenatal clinics over a period of about 30 months. Scheduled visits will be at Day 3, 7 and then every week until Day 63 post-treatment. A window period is allowed if study subjects are unable to attend on the scheduled date, i.e. ± 1-day for Day 7 and 14; ± 2 for Day 21 and 28; and ± 3 from Day 35 to Day 63. Women will be encouraged to attend the antenatal clinic between scheduled visits if sick.

Pregnant women recruited during the third trimester (before 37 weeks) may deliver before the end of the 63-day active follow up. In this case, the assessment at delivery will be done as planned but the active follow up will continue after delivery until Day 63. A blood sample to measure liver function test (LFT) and bilirubin will be taken within 48 hours of delivery from babies whose mothers delivered within 2 weeks of the inclusion in the trial.

The outcome of pregnancy, birth weight and maternal Hb will be collected as soon as possible after delivery. A placenta biopsy will be collected for later histopathological analysis. The new-born will be examined for congenital abnormalities. Both the mother and the new-born will be reassessed for any adverse event between 4 and 6 weeks and then after one year (only the baby). Patients will be assessed as summarized in the study visit schedule (Table 3).

Data collection and management

Plans for assessment and collection of outcomes

Study visits: At each visit, both scheduled and unscheduled, the medical history since the last visit (including any treatment taken), current signs and symptoms (if any) will be collected. A blood sample for thick smear will be collected and the body temperature checked. Dried blood spots for later genotyping to distinguish between recrudescence and new infection will be collected at Day 0, before treatment, and at every study visit. Information on any adverse event will also be collected. Haematology (full blood count) will be performed at Days 0, 7, 14, 28, 42 and 63; biochemistry (Total and conjugated bilirubin, AST, ALT, alkaline phosphatase, and creatinine) at Day 0, 1, and 7. In the event of increased liver function tests (LFTs) >3xULN, the result will be verified by taking an additional sample to be analysed (within 24 hours). Subsequent blood samples for LFTs will be taken at 48-hour intervals until the results return to <2xULN. ECG will be performed at Day 0, before drug intake, and at

For peer review only - http://bmjopen.bmj.com/site/about/guidelines.xhtml

Day 2 after drug intake. If abnormal at Day 2, ECG will be repeated at Day 7 and every week until return to normal. At the end of the active follow up, on Day 63, field assistants will visit the study subjects monthly to maintain the contact, but without collecting any data or biological samples.

Babies born from women who deliver within or 2 weeks after the active follow up will have a blood sample taken to measure LFT and bilirubin within 48 hours of delivery.

The outcome of pregnancy, birth weight and maternal Hb will be collected as soon as possible after delivery. A placenta biopsy will be collected, put immediately in 10% buffered formalin container stored in 4°C at the study site for later histopathological analysis. The new-born will be examined for congenital abnormalities. Both the mother and the new-born will be reassessed twice after delivery for any adverse event: between 4 and 6 weeks and then after one year.

PCR analysis

Genotyping of recurrent infections will be performed by characterizing the merozoites surface protein 1 (*msp1*), *msp2* and glutamate rich protein (*glurp)* genes, single-copy genes of the *P. falciparum* genome. PCR amplification of deoxyribonucleic acid (DNA) from a single parasite clone will yield a unique amplification product. For all three genes, each PCR amplification product of different size is considered from a different *P. falciparum* clone and reflects a different genotype. For samples collected from the same patient on Day 0 and on the Day of recurrent parasitaemia (after Day 3), the length polymorphism of *msp1*, *msp2* and *glurp* will be determined, i.e., the number of bands in each PCR reaction and their respective sizes. The results will be interpreted as follows:

Recurrence: at least one polymorphism of identical length for each marker (*msp1*, *msp2* and *glurp*) is found in the sample collected on Day 0 and on the Day of the recurrent parasitaemia.

New infection: For at least one marker, the length polymorphism is different between the sample collected on Day 0 and on the Day of recurrent parasitaemia.

Indeterminate: Samples that did not give a result due to an inability to amplify DNA on Day 0 and/or on the Day of recurrent parasitaemia.

Analysis of placental samples

The placental biopsy samples will be processed and embedded in paraffin wax using standard techniques. Paraffin sections 4 µm thick will be stained with haematoxylin-eosin. Placental biopsies after reading will be classified according to the following definitions (16): (i) acute

For peer review only - http://bmjopen.bmj.com/site/about/guidelines.xhtml

infection (parasite present, hemozoin absent or minimal deposition); (ii) chronic infection (parasites and heavy hemozoin deposition); (iii) past infection (no parasite but presence of hemozoin) or (iv) no infection (absence of parasites and hemozoin).

Haematological and biochemical analysis

Haematology (including haemoglobin) and biochemistry (including LFTs, i.e., AST, ALT, ALP, total and conjugated bilirubin) will be performed during active follow-up; haematology will be performed prior to the first dose of treatment, on Day 0, and then on Days 7, 14, 28, 42, and 63. An additional test will be performed at any unscheduled visit. In addition, only Hb will be measured at delivery. Biochemistry will also be done on Day 0, prior to treatment, then on Day 1 and Day 7. If LFTs increase more than 3 times the upper limit normal (ULN), the result will be verified by taking another sample for analysis as soon as possible (within 24 hours of the initial sample). Subsequent blood samples for LFTs will be taken at 48-hour intervals until results return to < 2xULN.

Statistical methods

Statistical methods for primary and secondary outcomes

The baseline characteristics will be described by treatment group and site. No statistical tests of significance will be undertaken, although the clinical significance of any imbalance will be noted.

The primary analysis will be the assessment of the non-inferiority of the PA compared to the DP and AL for the PCR-adjusted ACPR at Day 42. It will use the combined data from the five countries, with adjustment for any centre effect, using an additive model for response rates (i.e., a generalised linear model with a Bernoulli error distribution and an identity link function). This will allow the evaluation of two pairwise treatment comparisons, i.e., PA versus AL, PA versus DP.

Efficacy analysis (primary end point)

For the efficacy analysis, both a m-ITT approach and a PP approach will be adopted, with PP analysis being the main approach, as recommended for equivalence studies. The m-ITT population will include all participants who have received any amount of study drug and have confirmed *P. falciparum* infection prior treatment.

The PP population will consist of all participants meeting the following predefined criteria:

1. Fulfilling the entry criteria specified in the clinical study protocol.

For peer review only - http://bmjopen.bmj.com/site/about/guidelines.xhtml

2. Completed treatment, including not having vomited the study drug or, if vomited, received a repeat dose that was not vomited.

3. No previous or concomitant medication that would interfere with treatment outcome.

The PP population will be identified after locking the database, just before the statistical analysis.

For the primary endpoint, i.e. treatment efficacy at Day 42, the proportion of participants with PCR-adjusted ACPR will be determined by treatment arm. Similar procedures will be applied to the m-ITT population.

Safety analysis (secondary end point)

The safety population will include all participants randomised and treated with at least one dose of the three antimalarial treatments. Standard safety report tables summarize and list safety data. All AEs will be coded using the most recent version of the Medical Dictionary for Regulatory activities (MedDRA) dictionary. Treatment emergent AEs will be defined as all AEs which started after the first administration of study drug. These AEs will be summarized by primary system organ class and preferred term, separately for each treatment regimen and overall. Similar summaries will be provided for treatment emergent AEs considered to be related to study treatments. In addition, treatment emergent AEs will be summarized by primary system organ class, preferred term, and maximal severity.

Vital signs and routine safety laboratory data will be summarised descriptively by treatment regimen and overall, by time point. Absolute values and changes from baseline will be presented. Safety laboratory data will be classified according to the normal ranges (below, within, above) and summaries of changes from baseline in these categories will be provided by treatment regimen and overall. Further, safety laboratory values will be classified according to Common Terminology Criteria for Adverse Events (CTCAE) and shift tables of the baseline CTCAE category versus post baseline categories will be presented.

Pharmacokinetic analysis

PA availability in patients' blood will be compared between pregnant women infected and non-infected by HIV on day 0 before and 1 hour (hr), 2hr, 6hr and 10hr after the first PA dose. Additional comparison will be done on day 1 (24hr after the first PA dose while before the second PA dose), day 2 (before the last PA dose), day 7, 14, 21, 28 and 42.

For peer review only - http://bmjopen.bmj.com/site/about/guidelines.xhtml

Paper draft Version 10
November 6th , 2022

Study monitoring

The trial will be evaluated by monitors (a Clinical Research Organization will be contracted) (pre-study visit) for its preparedness to carry out, following by regular monitoring and closeout visits.

The coordination of the whole project is in the hand of the sponsor, wich will be the primary contact contact. He will be assisted by appropriate administrative and financial staff. A Coordinating Committee (CC) including one member of each institution will be main decision body of the consortium. For the trial, there will be three entities involved in its implementation and management, namely the Data Safety and Monitoring Board (DSMB), the Trial Steering Committee (TSC), and the Trial Management Group (TMG). Both DSMB and TSC are composed of independent experts to provide the overall supervision of he trial, monitor trial progress and advise on scientific credibility.

Ethics and dissemination

This protocol has been approved by Ethics Committee for Health Research (CERS) in Burkina Faso (Reference: 2020-3-047), the National Health Ethics Committee (CNES) in Democratic Republic of Congo (Reference: 169/CNES/BN/PMMF/2019), the Ethics Committee of the Faculty of Medicine and Odontostomatology (FMOS) / Faculty of Pharmacy (FPHA) in Mali (Reference: 2020/46/CE/FMOS/FAPH) in Mali, The Gambia Government/MRCG Joint Ethics Committee (Reference: 21818) and the National Bioethics Committee for Health (CNBS) in Mozambique (Reference: 313/CNBS/20).

Written informed consent will be taken from each individual prior to her participation in the study, the study investigators.

The outcomes of the project will be communicated to the National Malaria Control Programme of the respective countries and to the Global Malaria Programme of the World Health Organization. The results will be published in peer reviewed Open Access Journals and presented at (inter)national conferences and meetings.

A work package is focused on the development of a plan for internal and external project communication, on the development of communication tools, and on the dissemination and exploitation of the project's findings. All these activities will be in line with H2020 guidelines on dissemination and publication of results and will highlight the contribution of the EDCTP in tackling societal and health challenges.

Trial status

For peer review only - http://bmjopen.bmj.com/site/about/guidelines.xhtml

Paper draft Version 10
November 6th , 2022

Active recruitment

Abbreviations

ACPR	Adequate clinical and parasitological response
ACT	Artemisinin-based combination therapy
AE	Adverse event
AESI	Adverse event of particular interest
AL	Artemether-lumefantrine
ALP	Alkaline phosphatase
ALT	Alanine aminotransferase
AMC	Academic Medical Centre
AST	Aspartate aminotransferase
CC	Coordinating Committee
CISM	Centro de Investigação em Saúde da Manhiça
CRO	Contract Research Organization
CRUN	Clinical Research Unit of Nanoro
CTCAE	Common Terminology Criteria for Adverse Events
DNA	deoxyribonucleic acid
DP	Dihydroartemisinin-piperaquine
DSMB	Data Safety Monitoring Board
eCRF	Electronic case report form
EDCTP	European & Developing Countries Clinical Trials Partnership
ETF	Early treatment failure
FAPH	Faculty of Pharmacy
FMOS	Faculty of Medicine and Odontostomatology
GCP	Good Clinical Practice
glurp	Glutamate rich protein
HIV	Human immunodeficiency virus
HS-RDT	Highly sensitive rapid diagnostic test
IPTp-SP	Intermittent preventive treatment during pregnancy with sulfadoxine-pyrimethamine
IRSS	Institut de Recherche en Sciences de la Santé

For peer review only - http://bmjopen.bmj.com/site/about/guidelines.xhtml

MedDRA	Medical Dictionary for Regulatory activities
mITT	Modified Intention-to-treat
LFT	Liver Function Tests
LBW	Low birth weight
LCF	Late Clinical Failure
LLIN	Long-lasting insecticidal net
LPF	Late parasitological failure
LTF	Late treatment failure
MAS	Mefloquine-artesunate
MMV	Medicine of Malaria Venture
MRTC	Malaria Research and Training Center
MRCG	The MRC Unit The Gambia
msp1	Merozoites Surface Protein gene 1
NKI	Nederlands Kanker Institutu
NOAEL	No observed adverse effect level
P. falciparum	*Plasmodium falciparum*
P. vivax	*Plasmodium vivax*
PA	Pyronaridine-artesunate
PCR	Polymerase chain reaction
PP	Per protocol analysis
REDCap	Research Electronic Data Capture
SAE	Serious adverse event
SP	Sulfadoxine-pyrimethamine
TMG	Trial management group
TSC	Trial Steering Committee
ULN	Upper Limit Normal
UNIKIN	University of Kinshasa
USTTB	University of Sciences of Techniques and Technologies of Bamako
WANECAM	West African Network for Antimalarial Drugs
WHO	World Health Organization

Acknowledgments

For peer review only

Paper draft Version 10
November 6th , 2022

The consortium thanks Shinpoong Pharm.co., LTd for providing the study drug (pyronaridine-artesunate: Pyramax) and the Medicine of Malaria Venture (MMV) for technical support and assistance to this ongoing clinical trial. The project would like to thank patients and their advisors for their contribution to the study implementation.

Authors' information

Moussa Djimde: mdjimde@icermali.org

Japhet Kabalu Tshiongo: kabalujaphet@gmail.com

Hypolite Muhindo-Mavoko: hypomavoko@gmail.com

Halidou Tinto: halidoutinto@gmail.com

Esperanca Sevene: esevene68@gmail.com

Maminata Traoré: traoré_maminata@yahoo.fr

Anifa Vala: anifa.vala@manhica.net

Salesio Macuacua: salesio.macuacua@manhica.net

Bérenger Kaboré: kaboreberenger@gmail.com

Edgard Diniba Dabira: edabira@mrc.gm

Annette Erhart: Annette.Erhart@lshtm.ac.uk

Hamadoun Diakite: diakiteh@icermali.org

Mohamed Keita: medkeita@gmail.com

Mireia Piqueras: mireia.piqueras@isglobal.org

Raquel González: Raquel.gonzalez@isglobal.org

Clara Menéndez: Clara.menendez@isglobal.org

Thomas P. C. Dorlo: t.dorlo@nki.nl

Issaka Sagara: isagara@icermali.org

Petra Mens: p.f.mens@amsterdamumc.nl

Henk Schallig: h.d.schallig@amsterdamumc.nl

Umberto D'Alessandro: udalessandro@mrc.gm

Kassoum Kayentao: kayentao@icermali.org

Author's contributions

KK, UDA, HMM, HT, MT, ES, MP, RG, CM, TPCD, PFM and HDFHS were responsible for the conception and design of the clinical trial. MD and JKT wrote the first draft of the paper. All authors contributed to critical review and approved the final manuscript.

For peer review only - http://bmjopen.bmj.com/site/about/guidelines.xhtml

Paper draft Version 10
November 6th , 2022

Competing interests

None of the authors declare a conflict of interests.

Funding statement

This project is part of the EDCTP2 programme supported by the European Union (grant number RIA2017MC-2025-PYRAPREG).

REFRENCES

1. Takem EN, D'Alessandro U. Malaria in pregnancy. Mediterranean Journal of Hematology and Infectious Diseases. 2013.
2. Desai M, ter Kuile FO, Nosten F, McGready R, Asamoa K, Brabin B, et al. Epidemiology and burden of malaria in pregnancy. Lancet Infectious Diseases. 2007.
3. Rogerson SJ, Desai M, Mayor A, Sicuri E, Taylor SM, van Eijk AM. Burden, pathology, and costs of malaria in pregnancy: new developments for an old problem. The Lancet Infectious Diseases. 2018.
4. D'Alessandro U, Hill J, Tarning J, Pell C, Webster J, Gutman J, et al. Treatment of uncomplicated and severe malaria during pregnancy. The Lancet Infectious Diseases. 2018.
5. Dellicour S, Sevene E, McGready R, Tinto H, Mosha D, Manyando C, et al. First-trimester artemisinin derivatives and quinine treatments and the risk of adverse pregnancy outcomes in Africa and Asia: A meta-analysis of observational studies. PLoS Med. 2017;
6. Marrelli MT, Brotto M, Sharma H, Sarker S, Lambourne J, Fhogartaigh C, et al. The effect of malaria and anti-malarial drugs on skeletal and cardiac muscles. Malar J [Internet]. 2016;15(1):524. Available from: http://malariajournal.biomedcentral.com/articles/10.1186/s12936-016-1577-y
7. Meghna Desai, Jenny Hill, Silke Fernandes, Patrick Walker, Christopher Pell, Julie Gutman, Kassoum Kayentao, Raquel Gonzalez, Jayne Webster, Brian Greenwood, Michel Cot FOTK. Prevention of malaria in pregnancy. Lancet Infect Dis. 2018;18(4):e119(doi: 10.1016/S1473-3099(18)30064-1).
8. European Public Assessment Report (EPAR). Assessment report; Pyramax. https://www.ema.europa.eu/en/documents/medicine-outside-eu/pyramax-public-assessment-report_en.pdf; Accessed on 17 February 2021. 2016.

For peer review only - http://bmjopen.bmj.com/site/about/guidelines.xhtml

Paper draft Version 10
November 6th , 2022

9. Sagara I, Beavogui AH, Zongo I, Soulama I, Borghini-Fuhrer I, Fofana B, et al. Pyronaridine–artesunate or dihydroartemisinin–piperaquine versus current first-line therapies for repeated treatment of uncomplicated malaria: a randomised, multicentre, open-label, longitudinal, controlled, phase 3b/4 trial. Lancet. 2018;
10. D'Alessandro U. Existing antimalarial agents and malaria-treatment strategies. Expert Opinion on Pharmacotherapy. 2009.
11. Artemisinin-Based Treatments in Pregnant Women with Malaria. N Engl J Med. 2016;
12. Muhindo Mavoko H, Nabasumba C, Tinto H, D'Alessandro U, Grobusch MP, Lutumba P, et al. Impact of retreatment with an artemisinin-based combination on malaria incidence and its potential selection of resistant strains: study protocol for a randomized controlled clinical trial. Trials 2013 141 [Internet]. 2013 Sep 23 [cited 2021 Sep 17];14(1):1–15. Available from: https://trialsjournal.biomedcentral.com/articles/10.1186/1745-6215-14-307
13. Byakika-Kibwika P, Lamorde M, Mayanja-Kizza H, Merry C, Colebunders B, Van Geertruyden J-P. Therapeutics and Clinical Risk Management Update on the efficacy, effectiveness and safety of artemether-lumefantrine combination therapy for treatment of uncomplicated malaria infectious Diseases institute and infectious Diseases Network for Treatment and Research in Africa. Ther Clin Risk Manag [Internet]. 2010 [cited 2021 Sep 17];6–11. Available from: www.dovepress.com
14. Manyando C, Kayentao K, Dalessandro U, Okafor HU, Juma E, Hamed K. A systematic review of the safety and efficacy of artemether-lumefantrine against uncomplicated Plasmodium falciparum malaria during pregnancy. Malaria Journal. 2012.
15. Yan X, Su XG. Stratified Wilson and Newcombe Confidence Intervals for Multiple Binomial Proportions. Stat Biopharm Res. 2010;
16. Ismail MR, Ordi J, Menendez C, Ventura PJ, Aponte JJ, Kahigwa E, et al. Placental pathology in malaria: A histological, immunohistochemical, and quantitative study. Hum Pathol. 2000;
17. Kloprogge F, Workman L, Borrmann S, Tékété M, Lefèvre G, Hamed K, et al. Artemether-lumefantrine dosing for malaria treatment in young children and pregnant women: A pharmacokinetic-pharmacodynamic meta-analysis. PLoS Med [Internet]. 2018 Jun 1 [cited 2021 Jul 3];15(6). Available from: https://pubmed.ncbi.nlm.nih.gov/29894518/
18. Rijken MJ, McGready R, Phyo AP, Lindegardh N, Tarning J, Laochan N, et al.

For peer review only - http://bmjopen.bmj.com/site/about/guidelines.xhtml

Pharmacokinetics of dihydroartemisinin and piperaquine in pregnant and nonpregnant women with uncomplicated falciparum malaria. Antimicrob Agents Chemother [Internet]. 2011 Dec [cited 2021 Jul 3];55(12):5500–6. Available from: https://pubmed.ncbi.nlm.nih.gov/21947392/

19. Roth JM, Sawa P, Omweri G, Makio N, Osoti V, De Jong MD, et al. Molecular detection of residual parasitemia after pyronaridine-artesunate or artemether-lumefantrine treatment of uncomplicated plasmodium falciparum malaria in Kenyan children. Am J Trop Med Hyg [Internet]. 2018 Aug 13 [cited 2021 Jul 1];99(4):970–7. Available from: www.ajtmh.org.

20. Roth JM, Sawa P, Makio N, Omweri G, Osoti V, Okach S, et al. Pyronaridine-artesunate and artemether-lumefantrine for the treatment of uncomplicated Plasmodium falciparum malaria in Kenyan children: A randomized controlled non-inferiority trial NCT02411994 NCT. Malar J [Internet]. 2018 May 15 [cited 2021 Jul 1];17(1):199. Available from: https://doi.org/10.1186/s12936-018-2340-3

21. Rogerson SJ. Management of malaria in pregnancy [Internet]. Vol. 146, Indian Journal of Medical Research. Indian Council of Medical Research; 2017 [cited 2021 Jul 3]. p. 328–33. Available from: https://pubmed.ncbi.nlm.nih.gov/29355138/

22. Compaoré A, Dierickx S, Jaiteh F, Nahum A, Bohissou TFE, Tinto H, et al. Fear and rumours regarding placental biopsies in a malaria-in-pregnancy trial in Benin. Malar J [Internet]. 2018 Nov 15 [cited 2021 Jul 1];17(1):425. Available from: https://doi.org/10.1186/s12936-018-2578-9

23. Phyo AP, Ashley EA, Anderson TJC, Bozdech Z, Carrara VI, Sriprawat K, et al. Declining Efficacy of Artemisinin Combination Therapy Against P. Falciparum Malaria on the Thai-Myanmar Border (2003-2013): The Role of Parasite Genetic Factors. Clin Infect Dis [Internet]. 2016 Sep 15 [cited 2021 Jul 1];63(6):784–91. Available from: https://pubmed.ncbi.nlm.nih.gov/27313266/

24. Amaratunga C, Lim P, Suon S, Sreng S, Mao S, Sopha C, et al. Dihydroartemisinin-piperaquine resistance in Plasmodium falciparum malaria in Cambodia: A multisite prospective cohort study. Lancet Infect Dis [Internet]. 2016 Mar 1 [cited 2021 Jul 1];16(3):357–65. Available from: https://pubmed.ncbi.nlm.nih.gov/26774243/

25. Phuc BQ, Rasmussen C, Duong TT, Dong LT, Loi MA, Ménard D, et al. Treatment failure of dihydroartemisinin/piperaquine for plasmodium falciparum Malaria, Vietnam [Internet]. Vol. 23, Emerging Infectious Diseases. Centers for Disease Control and Prevention (CDC); 2017 [cited 2021 Jul 1]. p. 715–7. Available from:

For peer review only

For peer review only - http://bmjopen.bmj.com/site/about/guidelines.xhtml

https://pubmed.ncbi.nlm.nih.gov/28322709/

26. Mairet-Khedim M, Leang R, Marmai C, Khim N, Kim S, Ke S, et al. Clinical and In Vitro Resistance of Plasmodium falciparum to Artesunate-Amodiaquine in Cambodia . Clin Infect Dis [Internet]. 2020 May 27 [cited 2021 Jul 1]; Available from: https://academic.oup.com/cid/advance-article/doi/10.1093/cid/ciaa628/5847731

Appendices

Table legend

Table 2: Definition of early and late treatment failures

Table 3: Study visit schedule

Table 2: Definition of early and late treatment failures

**Early treatment failure (ETF)** one of the following	**Late treatment failure (LTF)**
1) Development of danger signs or severe malaria on Days 0-3 with parasitaemia<br><br>2) Presence of parasitaemia on Day 3 with fever (axillary temperature ≥ 37.5°C)	Late Clinical Failure (LCF):<br>1) Development of danger signs or severe malaria on any day after Day 3 in the presence of parasitaemia, without having previously met any of the ETF criteria;<br>2) Presence of parasitaemia and fever on any day after Day 3, without having previously met the ETF criteria.

For peer review only - http://bmjopen.bmj.com/site/about/guidelines.xhtml

	Late parasitological failure (LPF): Presence of parasitaemia on any day, starting on Day 7, and axillary temperature <37.5 ºC, without previously meeting any of the ETF or LCF criteria.

For peer review only

For peer review only - http://bmjopen.bmj.com/site/about/guidelines.xhtml

Paper draft Version 10
November 6th , 2022

Table 3: Study visit schedule

Day	0	1	2	3	7(± 1)	14 (± 1)	21 (± 2)	28 (± 2)	35 (± 3)	42 (± 3)	49-56 (± 3)	63 (± 3)	Any other day[1]	Delivery	4-6 weeks post-end of pregnancy	1-year post-end of pregnancy
History (symptoms)	X	X	X	X	X	X	X	X	X	X	X	X	X			
Informed consent	X															
Examination (clinical)	X	X	X	X	X	X	X	X	X	X	X	X	X	X		
Vital signs	X	X	X	X	X	X	X	X	X	X	X	X	X	X		
Foetal viability	X			X	X	X	X	X	X	X	X	X	X			
Blood Pressure	X	X	X	X	X	X	X	X	X	X	X	X	X	X		
Body Temperature	X	X	X	X	X	X	X	X	X	X	X	X	X	X		
ECG	X		X		X[2]											
Blood slide[3]	X	X	X	X	X	X	X	X	X	X	X	X	X	X		
Filter paper for genotyping	X	X	X	X	X	X	X	X	X	X	X	X	X	X		
Adverse drug reactions	X	X	X	X	X	X	X	X	X	X	X	X	X	X	X	
Concomitant medications	X	X	X	X	X	X	X	X	X	X	X	X	X	X	X	
Haematology	X				X	X		X		X		X	X	X[6]		
Treatment administration	X	X	X													
Blood sample for PK[4]	X	X	X		X	X	X	X		X						
Biochemistry[5]	X	X			X											
Urine analysis	X							X		X		X				
Placenta biopsy														X		
Newborn Assessment														X[8]	X	
Newborn LFT														X[7]		
Infant assessment																X

[1]Spontaneous attendance to the health facility (unscheduled visit); [2]ECG at Day 7 if abnormal at Day 2; [3]Thick and thin blood film; [4]Four blood samples at scheduled times, first sampling before treatment administration;[5] In the event of increased LFTs >3xULN, the result will be verified by taking a further sample for analysis as soon as possible (within 24 hours of the original sample). Subsequent blood samples for LFTs will be taken at 48-hour intervals until the results return to $\leq$2xULN;[6] Only Hb; [7]If delivery within or 2 weeks after the active follow up; [8]Blood sample collected systematically for dried blood spots, blood slide and Hb.

For peer review only - http://bmjopen.bmj.com/site/about/guidelines.xhtml

Paper draft Version 10
November 6th , 2022

Information sheet and Consent form

PARTICIPANT INFORMATION SHEET (WITHOUT PK COMPONENT)

Study Title:
Efficacy and Safety of Pyronaridine-Artesunate (PYRAMAX®) for the treatment of falciparum malaria in African pregnant women

Protocol: Version 1.5, 21st December 2020

Sponsor & Funder: University of Sciences, Techniques and Technologies of Bamako (USTTB), Mali; European & Developing Countries Clinical Trials Partnership (EDCTP)

What is informed consent?
You are invited to take part in a research study. Participating in a research study is not the same as getting regular medical care. The purpose of regular medical care is to improve one's health. The purpose of a research study is to gather information. It is your choice to take part and you can stop any time.

Before you decide you need to understand all information about this study and what it will involve. Please take time to read the following information or get the information explained to you in your language. Listen carefully and feel free to ask if there is anything that you do not understand. Ask for it to be explained until you are satisfied. You may also wish to consult your spouse, family members or others before deciding to take part in the study.

If you decide to join the study, you will need to sign or thumbprint a consent form saying you agree to be in the study.

Why is this study being done?
Malaria is a sickness caused by a very small parasite. This parasite can get into the body when a mosquito bites you. It can cause fevers, headaches, body aches, and weakness. If it is not treated, it can make a person very ill -- especially pregnant women. Your baby may also suffer from a malaria attack as babies born from malaria infected women are smaller, lack blood and have a higher chance of dying before and around delivery. Smaller babies are also at a high risk of dying before their first birthday. When malaria is treated with the right drugs, it can be cured. Some malaria parasites can become resistant to these drugs.
This research study is being done to learn more about the treatment of malaria in pregnancy. We want to compare different medicines for the treatment of mild malaria. The medicines we are studying are: pyronaridine-artesunate, artemether-lumefantrine and dihydroartemisinin-piperaquine. All of them are active against malaria but with this study we want to compare them when given to pregnant women with malaria. Two thousand three hundred and forty pregnant women from five different African countries will participate in this study. The results of the study will be made available to your community.

For peer review only - http://bmjopen.bmj.com/site/about/guidelines.xhtml

Paper draft Version 10
November 6th , 2022

What does this study involve?
You will be given by a study nurse one of the 3 medicines studied and the choice of the medicine will be made by chance. Two of these medicines are standard treatments for malaria in both pregnant and non-pregnant women with malaria. The other treatment, pyronaridine-artesunate, called also Pyramax, has been used in adults, including non-pregnant women, and children and is as effective as the other anti-malaria drugs. However, there is little information on its use in pregnant women. The doctor will follow you for about 2 months and also twice after the baby is born. You can choose to withdraw your consent to participate in the study at any time: in this case, you will be followed with routine antenatal and postnatal care in your country.

In case the investigator discovers you are sick and decides that you cannot participate in the study because of that, you will receive immediate care at the study site and then be referred to the appropriate health facility.

If you agree to participate in this study, we will ask you to **stay at the clinic today and attend the clinic the following 3 days** so that the doctor will be able to see if the medicine is working well. **You will receive medicine by mouth every day for the first three days. You will not have to pay for the treatment and care throughout the study. Please note that even if you decide that you do not want to be in this study, you will be treated for malaria, according to the protocol and procedures usually in place in your country. The doctor explaining this document to you will explain in more details the medicines used for malaria in your country. You will be treated with these medicines if you do not want to be in this study.**

Earlier, we will have taken a small amount of blood from your finger to see if you were eligible for this study. We would like to save some of this blood so that if the treatment does not work well and we have to give you another medicine, we can do some more tests on that blood to find out why the medicine does not work.

After the first 4 days, we will ask you to return one week after the beginning of treatment (Day 7), and thereafter every week until about 2 months from the first day of your treatment, at Day 63. **In addition, we will ask you to deliver in the local health facility and at that time we will check up you and your baby to make sure that the malaria has been cured. There will be 2 additional visits, one at around the time your baby is one month old and the other when your baby is 1 year old.** We will give you a calendar so that you know what days you should come.

A blood test to check if you have malaria will be done in the first 4 days and then again at every scheduled visit. Each time we do a blood test for malaria, we will put a few drops of blood onto a piece of paper. This blood will be used to learn about the malaria parasites that cause the infection. A test for lack of blood will be done on days 0, 7, 14, 28, 63 and then at delivery. We will also check the blood of the baby within 2 days from delivery, if possible immediately after, to check if he/she has malaria. We need to check your blood so often to make sure the treatment works.

At some visit we will collect an additional blood sample to measure the amount of blood you have and if your kidney and liver work well.

At each visit, we will examine you and ask some questions. If you do not come to the clinic, someone from the clinic will try to find you at home. At delivery, we will also prick your

For peer review only - http://bmjopen.bmj.com/site/about/guidelines.xhtml

baby's heel and take a few drops of blood to be sure your baby is not infected with malaria. We will examine your baby and make sure there are no problems from the drugs.

You will be visited at home 4-6 weeks after delivery and about 1 year after you have delivered, to check if your child is in good health.

If the malaria is not going away from your blood, we will give you another treatment. This will be either quinine tablets for 7 days or the standard treatment against malaria in your country. The treatment will be free of charge. We will also give you money for the transport to the clinic. Even if you choose to withdraw from the study, we would like to continue to follow you until the end of the 63 days to make sure that you are well. However, this is your decision.

If the research study needs to be stopped, you will be informed and you will have your normal medical care.

What will happen to the samples taken in this study?
The blood samples will be used to determine whether malaria parasites are present in your blood. In addition, we will check whether you have sufficient blood and also if your kidney and liver work well. We will also check if there is any abnormality in the urine. At delivery, we will collect a small piece of the placenta to check if malaria has attained it. These laboratory assays will be done in COUNTRY but some samples may be sent abroad to collaborators either for quality control or for additional tests. If you agree, any samples remaining will be stored at the laboratory of the sites. These samples may be used in the future for other research that aims to benefit people in Africa. These samples could be sent to laboratories abroad. We will not do genetic tests on these samples. Any future research would need to be approved by the local ethics committee in the same way this study has been approved. You can say "yes" or "no" to the other use of your samples. If you say "no", you can still be in the study. You can say "yes" now and change your mind later. Just tell us and we will destroy any of your remaining samples in storage. We will not contact you with the results of any future studies with your samples although these results could be provided to your community at meetings/open-days.

What harm or discomfort can you expect in the study?
You will have some discomfort and slight bruising when the blood sample is taken. Rarely people feel light-headed and may faint or there may be an infection, which is why we take the blood sample with you in a seated or lying position and disinfect the skin. The amount of blood removed will be too small to affect your health or that of your child. The drug we give you will treat your malaria. Malaria causes lack of blood. You are less likely to have lack of blood when the malaria is treated well.

There is little risk for your baby. Two of these drugs, artemether-lumefantrine and dihydroartemisinin-piperaquine, have been used in pregnant women before. They seem to be safe, but they may cause problems that we do not know about.

Side effects following treatment with the study medications could occur, namely nausea, headache, dizziness, bad dreams, bad sleeping. Generally, they are expected to be mild and short-lived. Rarely, more severe side effects can occur. If you have severe side effects, like persistent vomiting, severe skin or heart problems, we will stop the drug and you will be

For peer review only - http://bmjopen.bmj.com/site/about/guidelines.xhtml

treated free of charge. We will also recommend that you are treated with different malaria drugs in the future.

The other treatment, Pyramax, has been used in children and non-pregnant adults, but there is little information when used during pregnancy. We know that most common side effects seen with Pyramax have been headache, abdominal pain (stomach-ache) and vomiting. People who described these side effects mostly said that they were not severe.

When doing the usual health safety blood tests, an increase in some chemicals (called enzymes) has been found in some people that took Pyramax. These chemicals can indicate some damage to the liver, however, this did not make people feel unwell and no permanent damage to the liver was caused by this. If you experience unusual loss of appetite, nausea or tiredness, or gets dark-coloured urine or light-coloured stools or becomes yellow in the eyes, you should contact the study doctor straight away to tell them about this. If you know you have had liver problems before, you should tell the study doctor about this before you take part in the study.

In some people, anti-malaria drugs, including Pyramax, have also caused a drop in the number of oxygen-carrying cells in the blood. The number of these cells increases back to normal again after a short while.

Any side effects or other health issues occurring during the study will be followed up by the study doctor.

You will be monitored closely after receiving treatment for malaria with the study medications for any possible side effects of the drugs and will receive appropriate medical care for any problem that happens during the study.

Once you go back home, if you feel bad or your illness becomes worse you should return to the clinic right away, even if you have not been asked to return on that day. There will be a doctor at the clinic every day and every night who will give you treatment for these problems. You will not have to pay for any of the treatments you receive during the study, even on days when we did not tell you to come.

Return to the clinic if you:

- Have fever
- Have convulsions
- Are very sleepy/ tired
- Are vomiting everything
- Are unable to drink or eat
- Have a painful rash or mouth sores or red eyes
- Have chest pain or difficulty breathing or very fast breathing
- Have vaginal bleeding or painful cramps

What benefits can you expect in the study?
You will be treated for malaria and followed up carefully for about 2 months after treatment and you will not have to pay the service fee for any of your clinic visits during this study nor the medicines.

There will be someone here at the clinic every day. You may come for a visit at any time if you feel that you are ill, even on nights or weekends or in between visits.

For peer review only - http://bmjopen.bmj.com/site/about/guidelines.xhtml

Paper draft Version 10
November 6th , 2022

This study will also help us learn how much of the drug will work best in pregnant women. This may help you or someone you know in the future.

Will you be compensated for participating in the study?
You will not get paid for participation, but you will get either transport by XXX or get the costs for the transport reimbursed.

What happens if you refuse to participate in the study or change your mind later?
You are free to participate or not in the study and you have the right to stop participating at anytime without giving a reason. This will not affect the medical care that you would normally receive. In case you decide to withdraw your participation during the study, any information already generated from the samples until the time of withdrawal will be used and samples already collected, for which you have given consent, will also be analysed and data used. The study doctor may also ask for tests for your safety.

Should any new information become available during the study that may affect your participation, you will be informed as soon as possible.

If you are injured in the study what compensation will be available?
We will be responsible to provide for treatment caused by procedures of the research study.
If medical treatment is required as an emergency, please refer to your health centre or clinic and contact the field worker who gave his/her telephone number to you or contact Dr [Name] on [Phone number]. The sponsor of this study has taken an insurance policy to deal with possible injuries related to the study.

How will personal records remain confidential and who will have access to it?
All information that is collected about you in the course of the study will be kept strictly confidential. Your personal information will only be available to the study team members and might be seen by some rightful persons from the Ethics Committee, Government authorities and sponsor.

Who should you contact if you have questions?
If you have any queries or concerns you can contact Dr [Name] or Dr [Name] on [Phone number] and you can always call the personal numbers of the study staff given to you.
Please feel free to ask any question you might have about the research study.

Who has reviewed the study?
This study has been reviewed and approved by the [TO BE FILLED BY THE COUNTRY SITE] Ethics Committee, which consists of scientists and lay persons to protect your rights and wellbeing.

For peer review only - http://bmjopen.bmj.com/site/about/guidelines.xhtml

Paper draft Version 10
November 6th , 2022

CONSENT FORM

____
(Printed name of participant)

Subject Number: |__|__|__|__|__|__|

☐ I have read the written information and I: **or**

☐ I have had the information explained to me by study personnel in a language that I understand,

and I:

- confirm that my choice to participate is entirely voluntarily,
- confirm that I have had the opportunity to ask questions about this study and I am satisfied with the answers and explanations that have been given,
- understand that I grant access to data about me to authorised persons described in the information sheet,
- have received time to consider whether or not to take part in this study,
- agree to take part in this study.

Tick as appropriate

I agree for my samples to be shipped outside of The Gambia	Yes ☐	No ☐
I agree to further research on my samples as described in the information sheet	Yes ☐	No ☐

Participant's signature/ thumbprint*

Date (dd/mmm/yyyy) Time (24hr)

Printed name of witness*

Signature witness* Date (dd/mmm/yyyy) Time (24hr)

I attest that I have explained the study information accurately in ______________________ , and that the information was understood to the best of my knowledge by the participant and that she has freely given consent to participate *in the presence of the above named witness

For peer review only - http://bmjopen.bmj.com/site/about/guidelines.xhtml

(*Only required if the participant is unable to read and write).

Printed name of person obtaining consent ______________________________

Signature of person obtaining consent ______________________________

Date (dd/mmm/yyyy) Time (24hr)

** Only required if the participant is unable to read or write.*

A certified copy of the signed informed consent document must be provided to the participant

For peer review only - http://bmjopen.bmj.com/site/about/guidelines.xhtml

Paper draft Version 10
November 6th, 2022

PARTICIPANT INFORMATION SHEET (WITH PK COMPONENT)

Study Title:
Efficacy and Safety of Pyronaridine-Artesunate (PYRAMAX®) for the treatment of falciparum malaria in African pregnant women

Protocol: Version 1.5, 21st December 2020

Sponsor & Funder: University of Sciences, Techniques and Technologies of Bamako (USTTB), Mali; European & Developing Countries Clinical Trials Partnership (EDCTP)

What is informed consent?
You are invited to take part in a research study. Participating in a research study is not the same as getting regular medical care. The purpose of regular medical care is to improve one's health. The purpose of a research study is to gather information. It is your choice to take part and you can stop any time.

Before you decide you need to understand all information about this study and what it will involve. Please take time to read the following information or get the information explained to you in your language. Listen carefully and feel free to ask if there is anything that you do not understand. Ask for it to be explained until you are satisfied. You may also wish to consult your spouse, family members or others before deciding to take part in the study.

If you decide to join the study, you will need to sign or thumbprint a consent form saying you agree to be in the study.

Why is this study being done?
Malaria is a sickness caused by a very small parasite. This parasite can get into the body when a mosquito bites you. It can cause fevers, headaches, body aches, and weakness. If it is not treated, it can make a person very ill -- especially pregnant women. Your baby may also suffer from a malaria attack as babies born from malaria infected women are smaller, lack blood and have a higher chance of dying before and around delivery. Smaller babies are also at a high risk of dying before their first birthday. When malaria is treated with the right drugs, it can be cured. Some malaria parasites can become resistant to these drugs.
This research study is being done to learn more about the treatment of malaria in pregnancy. We want to compare different medicines for the treatment of mild malaria. The medicines we are studying are: pyronaridine-artesunate, artemether-lumefantrine and dihydroartemisinin-piperaquine. All of them are active against malaria but with this study we want to compare them when given to pregnant women with malaria. Two thousand three hundred and forty pregnant women from five different African countries will participate in this study. The results of the study will be made available to your community.

What does this study involve?
You will be given by a study nurse one of the 3 medicines studied and the choice of the medicine will be made by chance. Two of these medicines are standard treatments for malaria

For peer review only - http://bmjopen.bmj.com/site/about/guidelines.xhtml

in both pregnant and non-pregnant women with malaria. The other treatment, pyronaridine-artesunate, called also Pyramax, has been used in adults, including non-pregnant women, and children and is as effective as the other anti-malaria drugs. However, there is little information on its use in pregnant women. The doctor will follow you for about 2 months and also twice after the baby is born. You can choose to withdraw your consent to participate in the study at any time: in this case, you will be followed with routine antenatal and postnatal care in your country.

In case the investigator discovers you are sick and decides that you cannot participate in the study because of that, you will receive immediate care at the study site and then be referred to the appropriate health facility.

If you agree to participate in this study, we will ask you to **stay at the clinic today and attend the clinic the following 3 days** so that the doctor will be able to see if the medicine is working well. **You will receive medicine by mouth every day for the first three days. You will not have to pay for the treatment and care throughout the study. Please note that even if you decide that you do not want to be in this study, you will be treated for malaria, according to the protocol and procedures usually in place in your country. The doctor explaining this document to you will explain in more details the medicines used for malaria in your country. You will be treated with these medicines if you do not want to be in this study.**

Earlier, we will have taken a small amount of blood from your finger to see if you were eligible for this study. We would like to save some of this blood so that if the treatment does not work well and we have to give you another medicine, we can do some more tests on that blood to find out why the medicine does not work.

To know whether we are giving the right amount of treatment, we would like to know how much treatment is in your blood. For this, we will collect some blood samples before treatment and then after treatment. Each time we will collect the blood from your vein but the quantity would be a few drops. The first day of treatment, besides the sample collected before treatment, we will collect 4 additional samples at different hours. We will collect another sample the following 2 days and then another 5 times until 1 month and 2 weeks after you have started treatment. This blood will be sent to another laboratory abroad for the analysis. If you do not want to participate to this specific component of the study, you can refuse but still be included in the research study for the other activities.

After the first 4 days, we will ask you to return one week after the beginning of treatment (Day 7), and thereafter every week until about 2 months from the first day of your treatment, at Day 63. **In addition, we will ask you to deliver in the local health facility and at that time we will check up you and your baby to make sure that the malaria has been cured. There will be 2 additional visits, one at around the time your baby is one month old and the other when your baby is 1 year old.** We will give you a calendar so that you know what days you should come.

A blood test to check if you have malaria will be done in the first 4 days and then again at every scheduled visit. Each time we do a blood test for malaria, we will put a few drops of blood onto a piece of paper. This blood will be used to learn about the malaria parasites that cause the infection. A test for lack of blood will be done on days 0, 7, 14, 28, 63 and then at

For peer review only - http://bmjopen.bmj.com/site/about/guidelines.xhtml

delivery. We will also check the blood of the baby within 2 days from delivery, if possible immediately after, to check if he/she has malaria. We need to check your blood so often to make sure the treatment works.

At some visit we will collect an additional blood sample to measure the amount of blood you have and if your kidney and liver work well.

At each visit, we will examine you and ask some questions. If you do not come to the clinic, someone from the clinic will try to find you at home. At delivery, we will also prick your baby's heel and take a few drops of blood to be sure your baby is not infected with malaria. We will examine your baby and make sure there are no problems from the drugs.

You will be visited at home 4-6 weeks after delivery and about 1 year after you have delivered, to check if your child is in good health.

If the malaria is not going away from your blood, we will give you another treatment. This will be either quinine tablets for 7 days or the standard treatment against malaria in your country. The treatment will be free of charge. We will also give you money for the transport to the clinic. Even if you choose to withdraw from the study, we would like to continue to follow you until the end of the 63 days to make sure that you are well. However, this is your decision.

If the research study needs to be stopped, you will be informed and you will have your normal medical care.

What will happen to the samples taken in this study?
The blood samples will be used to determine whether malaria parasites are present in your blood. In addition, we will check whether you have sufficient blood and also if your kidney and liver work well. We will also check if there is any abnormality in the urine. At delivery, we will collect a small piece of the placenta to check if malaria has attained it. These laboratory assays will be done in the country but some samples may be sent abroad to collaborators either for quality control or for additional tests. If you agree, any samples remaining will be stored at the in the labs. These samples may be used in the future for other research that aims to benefit people in Africa. These samples could be sent to laboratories abroad. We will not do genetic tests on these samples. Any future research would need to be approved by the local ethics committee in the same way this study has been approved. You can say "yes" or "no" to the other use of your samples. If you say "no", you can still be in the study. You can say "yes" now and change your mind later. Just tell us and we will destroy any of your remaining samples in storage. We will not contact you with the results of any future studies with your samples although these results could be provided to your community at meetings/open-days.

What harm or discomfort can you expect in the study?
You will have some discomfort and slight bruising when the blood sample is taken. Rarely people feel light-headed and may faint or there may be an infection, which is why we take the blood sample with you in a seated or lying position and disinfect the skin. The amount of blood removed will be too small to affect your health or that of your child. The drug we give you will treat your malaria. Malaria causes lack of blood. You are less likely to have lack of blood when the malaria is treated well.

For peer review only - http://bmjopen.bmj.com/site/about/guidelines.xhtml

Paper draft Version 10
November 6th , 2022

There is little risk for your baby. Two of these drugs, artemether-lumefantrine and dihydroartemisinin-piperaquine, have been used in pregnant women before. They seem to be safe, but they may cause problems that we do not know about.

Side effects following treatment with the study medications could occur, namely nausea, headache, dizziness, bad dreams, bad sleeping. Generally, they are expected to be mild and short-lived. Rarely, more severe side effects can occur. If you have severe side effects, like persistent vomiting, severe skin or heart problems, we will stop the drug and you will be treated free of charge. We will also recommend that you are treated with different malaria drugs in the future.

The other treatment, Pyramax, has been used in children and non-pregnant adults, but there is little information when used during pregnancy. We know that most common side effects seen with Pyramax have been headache, abdominal pain (stomach-ache) and vomiting. People who described these side effects mostly said that they were not severe.

When doing the usual health safety blood tests, an increase in some chemicals (called enzymes) has been found in some people that took Pyramax. These chemicals can indicate some damage to the liver, however, this did not make people feel unwell and no permanent damage to the liver was caused by this. If you experience unusual loss of appetite, nausea or tiredness, or gets dark-coloured urine or light-coloured stools or becomes yellow in the eyes, you should contact the study doctor straight away to tell them about this. If you know you have had liver problems before, you should tell the study doctor about this before you take part in the study.

In some people, anti-malaria drugs, including Pyramax, have also caused a drop in the number of oxygen-carrying cells in the blood. The number of these cells increases back to normal again after a short while.

Any side effects or other health issues occurring during the study will be followed up by the study doctor.

You will be monitored closely after receiving treatment for malaria with the study medications for any possible side effects of the drugs and will receive appropriate medical care for any problem that happens during the study.

Once you go back home, if you feel bad or your illness becomes worse you should return to the clinic right away, even if you have not been asked to return on that day. There will be a doctor at the clinic every day and every night who will give you treatment for these problems. You will not have to pay for any of the treatments you receive during the study, even on days when we did not tell you to come.

Return to the clinic if you:

- Have fever
- Have convulsions
- Are very sleepy/ tired
- Are vomiting everything
- Are unable to drink or eat
- Have a painful rash or mouth sores or red eyes
- Have chest pain or difficulty breathing or very fast breathing
- Have vaginal bleeding or painful cramps

For peer review only - http://bmjopen.bmj.com/site/about/guidelines.xhtml

Paper draft Version 10
November 6th , 2022

What benefits can you expect in the study?
You will be treated for malaria and followed up carefully for about 2 months after treatment and you will not have to pay the service fee for any of your clinic visits during this study nor the medicines.

There will be someone here at the clinic every day. You may come for a visit at any time if you feel that you are ill, even on nights or weekends or in between visits.

This study will also help us learn how much of the drug will work best in pregnant women. This may help you or someone you know in the future.

Will you be compensated for participating in the study?
You will not get paid for participation, but you will get either transport by XXX or get the costs for the transport reimbursed.

What happens if you refuse to participate in the study or change your mind later?
You are free to participate or not in the study and you have the right to stop participating at anytime without giving a reason. This will not affect the medical care that you would normally receive. In case you decide to withdraw your participation during the study, any information already generated from the samples until the time of withdrawal will be used and samples already collected, for which you have given consent, will also be analysed and data used. The study doctor may also ask for tests for your safety.

Should any new information become available during the study that may affect your participation, you will be informed as soon as possible.

If you are injured in the study what compensation will be available?
We will be responsible to provide for treatment caused by procedures of the research study.
If medical treatment is required as an emergency, please refer to your health centre or clinic and contact the field worker who gave his/her telephone number to you or contact Dr [Name] on [Phone number]. The sponsor of this study has taken an insurance policy to deal with possible injuries related to the study.

How will personal records remain confidential and who will have access to it?
All information that is collected about you in the course of the study will be kept strictly confidential. Your personal information will only be available to the study team members and might be seen by some rightful persons from the Ethics Committee, Government authorities and sponsor.

Who should you contact if you have questions?
If you have any queries or concerns you can contact Dr [Name] or Dr [Name] on [Phone number] and you can always call the personal numbers of the study staff given to you.
Please feel free to ask any question you might have about the research study.

Who has reviewed the study?

For peer review only - http://bmjopen.bmj.com/site/about/guidelines.xhtml

Paper draft Version 10
November 6th , 2022

This study has been reviewed and approved by the [TO BE FILLED BY THE COUNTRY SITE] Ethics Committee, which consists of scientists and lay persons to protect your rights and wellbeing.

For peer review only

For peer review only - http://bmjopen.bmj.com/site/about/guidelines.xhtml

Paper draft Version 10
November 6th , 2022

CONSENT FORM

____

(Printed name of participant)

Subject Number: |__|__|__|__|__|

☐ I have read the written information and I: **or**

☐ I have had the information explained to me by study personnel in a language that I understand,

and I:

- confirm that my choice to participate is entirely voluntarily,
- confirm that I have had the opportunity to ask questions about this study and I am satisfied with the answers and explanations that have been given,
- understand that I grant access to data about me to authorised persons described in the information sheet,
- have received time to consider whether or not to take part in this study,
- agree to take part in this study.

Tick as appropriate

I agree to be part of the pharmacokinetic study	Yes ☐	No ☐
I agree for my samples to be shipped outside of The Gambia	Yes ☐	No ☐
I agree to further research on my samples as described in the information sheet	Yes ☐	No ☐

Participant's signature/ thumbprint* ☐

____

Date (dd/mmm/yyyy) Time (24hr)

Printed name of witness*

____

____

Signature witness* Date (dd/mmm/yyyy) Time (24hr)

For peer review only - http://bmjopen.bmj.com/site/about/guidelines.xhtml

Paper draft Version 10
November 6th , 2022

I attest that I have explained the study information accurately in ______________________ , and that the information was understood to the best of my knowledge by the participant and that she has freely given consent to participate *in the presence of the above named witness (*Only required if the participant is unable to read and write).

Printed name of person obtaining consent ____

Signature of person obtaining consent ____
Date (dd/mmm/yyyy) Time (24hr)

** Only required if the participant is unable to read or write.*

A certified copy of the signed informed consent document must be provided to the participant

For peer review only - http://bmjopen.bmj.com/site/about/guidelines.xhtml

SPIRIT — STANDARD PROTOCOL ITEMS: RECOMMENDATIONS FOR INTERVENTIONAL TRIALS

SPIRIT 2013 Checklist: Recommended items to address in a clinical trial protocol and related documents*

Section/item	Item No	Description	Addressed on page number
**Administrative information**			
Title	1	Descriptive title identifying the study design, population, interventions, and, if applicable, trial acronym	1
Trial registration	2a	Trial identifier and registry name. If not yet registered, name of intended registry	2
	2b	All items from the World Health Organization Trial Registration Data Set	
Protocol version	3	Date and version identifier	2
Funding	4	Sources and types of financial, material, and other support	18-19
Roles and responsibilities	5a	Names, affiliations, and roles of protocol contributors	18
	5b	Name and contact information for the trial sponsor	2
	5c	Role of study sponsor and funders, if any, in study design; collection, management, analysis, and interpretation of data; writing of the report; and the decision to submit the report for publication, including whether they will have ultimate authority over any of these activities	2
	5d	Composition, roles, and responsibilities of the coordinating centre, steering committee, endpoint adjudication committee, data management team, and other individuals or groups overseeing the trial, if applicable (see Item 21a for data monitoring committee)	2

For peer review only

**Introduction**			
Background and rationale	6a	Description of research question and justification for undertaking the trial, including summary of relevant studies (published and unpublished) examining benefits and harms for each intervention	__5_________
	6b	Explanation for choice of comparators	___5, 9-10______
Objectives	7	Specific objectives or hypotheses	____5,6_______
Trial design	8	Description of trial design including type of trial (eg, parallel group, crossover, factorial, single group), allocation ratio, and framework (eg, superiority, equivalence, noninferiority, exploratory)	_____5,7_______
**Methods: Participants, interventions, and outcomes**			
Study setting	9	Description of study settings (eg, community clinic, academic hospital) and list of countries where data will be collected. Reference to where list of study sites can be obtained	_____8________
Eligibility criteria	10	Inclusion and exclusion criteria for participants. If applicable, eligibility criteria for study centres and individuals who will perform the interventions (eg, surgeons, psychotherapists)	____8-9________
Interventions	11a	Interventions for each group with sufficient detail to allow replication, including how and when they will be administered	__9-10________
	11b	Criteria for discontinuing or modifying allocated interventions for a given trial participant (eg, drug dose change in response to harms, participant request, or improving/worsening disease)	____9-10_______
	11c	Strategies to improve adherence to intervention protocols, and any procedures for monitoring adherence (eg, drug tablet return, laboratory tests)	____8-9,12-13___
	11d	Relevant concomitant care and interventions that are permitted or prohibited during the trial	____9_________
Outcomes	12	Primary, secondary, and other outcomes, including the specific measurement variable (eg, systolic blood pressure), analysis metric (eg, change from baseline, final value, time to event), method of aggregation (eg, median, proportion), and time point for each outcome. Explanation of the clinical relevance of chosen efficacy and harm outcomes is strongly recommended	__10-11_______
Participant timeline	13	Time schedule of enrolment, interventions (including any run-ins and washouts), assessments, and visits for participants. A schematic diagram is highly recommended (see Figure)	_____12, 25__

Sample size	14	Estimated number of participants needed to achieve study objectives and how it was determined, including clinical and statistical assumptions supporting any sample size calculations	_11____________
Recruitment	15	Strategies for achieving adequate participant enrolment to reach target sample size	__9, 12________

Methods: Assignment of interventions (for controlled trials)

Allocation:

Sequence generation	16a	Method of generating the allocation sequence (eg, computer-generated random numbers), and list of any factors for stratification. To reduce predictability of a random sequence, details of any planned restriction (eg, blocking) should be provided in a separate document that is unavailable to those who enrol participants or assign interventions	___9__________
Allocation concealment mechanism	16b	Mechanism of implementing the allocation sequence (eg, central telephone; sequentially numbered, opaque, sealed envelopes), describing any steps to conceal the sequence until interventions are assigned	______9_______
Implementation	16c	Who will generate the allocation sequence, who will enrol participants, and who will assign participants to interventions	_______9______
Blinding (masking)	17a	Who will be blinded after assignment to interventions (eg, trial participants, care providers, outcome assessors, data analysts), and how	________9_____
	17b	If blinded, circumstances under which unblinding is permissible, and procedure for revealing a participant's allocated intervention during the trial	______8_______

Methods: Data collection, management, and analysis

Data collection methods	18a	Plans for assessment and collection of outcome, baseline, and other trial data, including any related processes to promote data quality (eg, duplicate measurements, training of assessors) and a description of study instruments (eg, questionnaires, laboratory tests) along with their reliability and validity, if known. Reference to where data collection forms can be found, if not in the protocol	_____12-13_____
	18b	Plans to promote participant retention and complete follow-up, including list of any outcome data to be collected for participants who discontinue or deviate from intervention protocols	______12-13___

Data management	19	Plans for data entry, coding, security, and storage, including any related processes to promote data quality (eg, double data entry; range checks for data values). Reference to where details of data management procedures can be found, if not in the protocol	_______12-13____
Statistical methods	20a	Statistical methods for analysing primary and secondary outcomes. Reference to where other details of the statistical analysis plan can be found, if not in the protocol	_____14-15______
	20b	Methods for any additional analyses (eg, subgroup and adjusted analyses)	___14-15_______
	20c	Definition of analysis population relating to protocol non-adherence (eg, as randomised analysis), and any statistical methods to handle missing data (eg, multiple imputation)	_____15__________
**Methods: Monitoring**			
Data monitoring	21a	Composition of data monitoring committee (DMC); summary of its role and reporting structure; statement of whether it is independent from the sponsor and competing interests; and reference to where further details about its charter can be found, if not in the protocol. Alternatively, an explanation of why a DMC is not needed	_____16__________
	21b	Description of any interim analyses and stopping guidelines, including who will have access to these interim results and make the final decision to terminate the trial	_____14_____
Harms	22	Plans for collecting, assessing, reporting, and managing solicited and spontaneously reported adverse events and other unintended effects of trial interventions or trial conduct	___14______
Auditing	23	Frequency and procedures for auditing trial conduct, if any, and whether the process will be independent from investigators and the sponsor	_____16__________
**Ethics and dissemination**			
Research ethics approval	24	Plans for seeking research ethics committee/institutional review board (REC/IRB) approval	______16_________
Protocol amendments	25	Plans for communicating important protocol modifications (eg, changes to eligibility criteria, outcomes, analyses) to relevant parties (eg, investigators, REC/IRBs, trial participants, trial registries, journals, regulators)	__16____________

For peer review only - http://bmjopen.bmj.com/site/about/guidelines.xhtml

Consent or assent	26a	Who will obtain informed consent or assent from potential trial participants or authorised surrogates, and how (see Item 32)	___16__________
	26b	Additional consent provisions for collection and use of participant data and biological specimens in ancillary studies, if applicable	____NA________ _
Confidentiality	27	How personal information about potential and enrolled participants will be collected, shared, and maintained in order to protect confidentiality before, during, and after the trial	____16_________
Declaration of interests	28	Financial and other competing interests for principal investigators for the overall trial and each study site	_____20_________
Access to data	29	Statement of who will have access to the final trial dataset, and disclosure of contractual agreements that limit such access for investigators	___9.13. 17____
Ancillary and post-trial care	30	Provisions, if any, for ancillary and post-trial care, and for compensation to those who suffer harm from trial participation	_______NA____
Dissemination policy	31a	Plans for investigators and sponsor to communicate trial results to participants, healthcare professionals, the public, and other relevant groups (eg, via publication, reporting in results databases, or other data sharing arrangements), including any publication restrictions	___16__________
	31b	Authorship eligibility guidelines and any intended use of professional writers	_____None_____
	31c	Plans, if any, for granting public access to the full protocol, participant-level dataset, and statistical code	_____None_____
**Appendices**			
Informed consent materials	32	Model consent form and other related documentation given to participants and authorised surrogates	_____26-40____
Biological specimens	33	Plans for collection, laboratory evaluation, and storage of biological specimens for genetic or molecular analysis in the current trial and for future use in ancillary studies, if applicable	_____12-14____

*It is strongly recommended that this checklist be read in conjunction with the SPIRIT 2013 Explanation & Elaboration for important clarification on the items. Amendments to the protocol should be tracked and dated. The SPIRIT checklist is copyrighted by the SPIRIT Group under the Creative Commons "Attribution-NonCommercial-NoDerivs 3.0 Unported" license.

For peer review only - http://bmjopen.bmj.com/site/about/guidelines.xhtml

BMJ Open

Efficacy and safety of pyronaridine-artesunate (PYRAMAX□) for the treatment of P. falciparum uncomplicated malaria in African pregnant women (PYRAPREG): study protocol for a phase 3, non-inferiority, randomized open-label clinical trial

Journal:	*BMJ Open*
Manuscript ID	bmjopen-2022-065295.R1
Article Type:	Protocol
Date Submitted by the Author:	17-Apr-2023
Complete List of Authors:	Djimde, Moussa; University of Sciences Techniques and Technologies of Bamako, Malaria Research and Training Center<br>Tshiongo, Japhet ; Universite de Kinshasa, Départment of Tropical Médecine<br>Muhindo, Hypolite ; Universite de Kinshasa Faculte de Medecine, Department of Tropical Medicine<br>Tinto, Halidou; Institut de Recherche en Sciences de la Santé (IRSS) – Unité de Recherche Clinique de Nanoro, Burkina Faso Ouagadougou, BF<br>Sevene, Esperanca; Centro de Investigacao em Saude de Manhica; Universidade Eduardo Mondlane<br>Traore, Maminata; Institut de Recherche en Sciences de la Santé (IRSS) – Unité de Recherche Clinique de Nanoro, Burkina Faso<br>Vala, Anifa; Centro de Investigação em Saúde de Manhiça<br>Macuacua, Salesio; Centro de Investigação em Saúde da Manhiça (CISM), Mozambique<br>Kabore, Berenger; Institut de Recherche en Sciences de la Santé (IRSS) – Unité de Recherche Clinique de Nanoro, Burkina Faso<br>Dabira, Edgard; MRC Unit The Gambia (MRCG) at the London School of Hygiene and Tropical Medicine, The Gambia London, UK<br>Erhart, Annette; MRC Unit The Gambia (MRCG) at the London School of Hygiene and Tropical Medicine, The Gambia<br>Diakite, Hamadoun ; University of Sciences Techniques and Technologies of Bamako, Malaria Research & Training Centre<br>Keita, Mohamed; University of Sciences Techniques and Technologies of Bamako Faculty of Medicine Odontostomatology<br>Piqueras, Mireia; Instituto de Salud Global Barcelona Barcelona, Catalunya, ES<br>González, Raquel; Instituto de Salud Global Barcelona Barcelona, Catalunya, ES<br>Menendez, Clara; Instituto de Salud Global Barcelona Barcelona, Catalunya, ES Barcelona, ES<br>Dorlo, Thomas; Netherlands Cancer Institute / Antoni van Leeuwenhoek Hospital, Amsterdam, the Netherlands<br>Sagara, Issaka; Universite des Sciences des Techniques et des Technologies de Bamako, Malaria Research and Training Center (MRTC)<br>Mens, Petra; Amsterdam University Medical Centres, Academic Medical Centre at the University of Amsterdam (AMC), Amsterdam, Netherlands<br>Schallig, Henk; Amsterdam University Medical Centres, Academic Medical

	Centre at the University of Amsterdam (AMC), Amsterdam, Netherlands<br>D'Alessandro, Umberto; MRC Laboratories The Gambia<br>Kayentao, Kassoum; University of Sciences Techniques and Technologies of Bamako, Malaria Research & Training Centre
**Primary Subject Heading**:	Epidemiology
Secondary Subject Heading:	Infectious diseases
Keywords:	EPIDEMIOLOGY, Infection control < INFECTIOUS DISEASES, Tropical medicine < INFECTIOUS DISEASES, PARASITOLOGY

SCHOLARONE™
Manuscripts

Paper draft Version 11
April 12th, 2023

Administrative information

Efficacy and safety of pyronaridine-artesunate (PYRAMAX®) for the treatment of *P. falciparum* uncomplicated malaria in African pregnant women (PYRAPREG): study protocol for a phase 3, non-inferiority, randomized open-label clinical trial

Moussa Djimde[1,2¥], Japhet Kabalu Tshiongo[2,3¥], Hypolite Muhindo Mavoko[3], Halidou Tinto[4], Esperanca Sevene[5,6], Maminata Traoré[4], Anifa Vala[5], Salesio Macuacua[5], Bérenger Kaboré[4], Edgard Diniba Dabira[7], Annette Erhart[7], Hamadoun Diakité[1], Mohamed Keita[1], Mireia Piqueras[8], Raquel González[5,8], Clara Menéndez[5,8], Thomas P.C. Dorlo[9], Issaka Sagara[1], Petra F. Mens[2], Henk D. F. H. Schallig[2], Umberto D'Alessandro[7] and Kassoum Kayentao[1*]

[¥] These authors contributed equally

Author's Affiliations:

[1]Malaria Research and Training Center (MRTC), University of Sciences of Techniques and Technologies of Bamako (USTTB), Mali

[2]Amsterdam University Medical Centres, Academic Medical Centre at the University of Amsterdam (AMC), Amsterdam, Netherlands

[3]Department of Tropical Medicine, University of Kinshasa (UNIKIN), Kinshasa, Democratic Republic of the Congo

[4]Institut de Recherche en Sciences de la Santé (IRSS) – Unité de Recherche Clinique de Nanoro, Burkina Faso

[5]Centro de Investigação em Saúde da Manhiça (CISM), Mozambique

[6]Faculty of Medicine, Eduardo Mondlane University, Mozambique

[7]MRC Unit The Gambia (MRCG) at the London School of Hygiene and Tropical Medicine, The Gambia

[8]Barcelona Institute for Global Health (ISGlobal), Hospital Clínic-Universitat de Barcelona, Spain

[9] Netherlands Cancer Institute / Antoni van Leeuwenhoek Hospital, Amsterdam, the Netherlands

For peer review only - http://bmjopen.bmj.com/site/about/guidelines.xhtml

Paper draft Version 11
April 12th, 2023

Trial registration:

PACTR202011812241529

Protocol version: 7.0 12 July 2022

Trial Sponsor and role

The trial sponsor is University of Sciences, Techniques, and Technologies of Bamako (USTTB). Hamdallaye ACI 2000, Rue 405 - Porte 359, Bamako

Téléphone : +223 20 29 04 07

http://usttb.edu.ml/

The Trial sponsor in the study has the responsibility to ensure that data and source documents granted for trial monitoring, audits, DSMB and Ethics Committee review and regulatory inspections as appropriate.

***Corresponding Author**: Kassoum Kayentao (kayentao@icermali.org)

Malaria Research and Training Center (MRTC), University of Sciences of Techniques and Technologies of Bamako (USTTB), Mali

For peer review only

For peer review only - http://bmjopen.bmj.com/site/about/guidelines.xhtml

Paper draft Version 11
April 12th, 2023

Abstract

Introduction: Malaria infection during pregnancy increases the risk of low birthweight and infant mortality and should be prevented and treated. Artemisinin-based combination treatments are generally well tolerated, safe and effective, the most used being artemether-lumefantrine (AL) and dihydroartemisinin-piperaquine (DP). Pyronaridine-artesunate (PA) is a new artemisinin-based combination. The main objective of this study is to determine the efficacy and safety of PA versus AL or DP when administered to pregnant women with confirmed *P. falciparum* infection in the second or third trimester. The primary hypothesis is the pair-wise non-inferiority of PA as compared to either AL or DP.

Methods and analysis: A phase 3, non-inferiority, randomized, open-label clinical trial to determine the safety and efficacy of AL, DP, and PA in pregnant women with malaria in 5 sub-Saharan, malaria-endemic countries (Burkina Faso, Democratic Republic of the Congo, Mali, Mozambique and The Gambia). A total of 1,875 pregnant women will be randomized to one of the treatment arms. Women will be actively monitored until the Day 63 post-treatment, at delivery and 4-6 weeks after delivery, and infants' health will be checked at their first birthday. The primary endpoint is the PCR-adjusted rate of adequate clinical and parasitological response at Day 42 in the per protocol population.

Ethics and Dissemination: This protocol has been approved by Ethics Committee for Health Research in Burkina Faso, the National Health Ethics Committee in Democratic Republic of Congo, the Ethics Committee of the Faculty of Medicine and Odontostomatology / Faculty of Pharmacy in Mali in Mali, The Gambia Government/MRCG Joint Ethics Committee and the National Bioethics Committee for Health in Mozambique. Written informed consent will be obtained from each individual prior to her participation in the study. The results will be published in peer reviewed Open Access Journals and presented at (inter)national conferences and meetings.

Keywords:

Malaria, pregnant women, pyronaridine-artesunate, artemether-lumefantrine, dihydroartemisinin-piperaquine, , Burkina Faso, Democratic Republic of the Congo, Mali, Mozambique, The Gambia.

For peer review only - http://bmjopen.bmj.com/site/about/guidelines.xhtml

Paper draft Version 11
April 12th, 2023

Strengths and limitations of this study

- This is randomized clinical trial that will provide evidence on the safety and efficacy of a newly registered Artemisinin-Based Combination pyronaridine-artesunate (PA) for the treatment of uncomplicated malaria in African pregnant women during the second and third trimester of pregnancy.
- The trial will generate additional data on the safety and efficacy of dihydroartemisinin-piperaquine, and artemether-lumefantrine and will increase substantially the treatment options for malaria in pregnancy.
- As PA is the last Artemisinin-Based Combination to have been registered for the treatment of malaria, the proposed trial will complement the information already available for malaria treatment in children and non-pregnant adults.
- Weaknesses include the lack of regular monitoring of children within the first year of birth and the non-inclusion of infected women in the first trimester.

For peer review only

For peer review only - http://bmjopen.bmj.com/site/about/guidelines.xhtml

Paper draft Version 11
April 12th, 2023

INTRODUCTION

Pregnancy is associated with hormonal changes and reduced immunity that make pregnant women more vulnerable to malaria [1]. Malaria during pregnancy increases the risk of low birth weight (LBW) which is associated with higher infant mortality.[2] The prevention and control of malaria during pregnancy is based on insecticide-treated nets (ITNs), intermittent preventive treatment during pregnancy (IPTp) with sulfadoxine-pyrimethamine (SP), and effective case management of clinical malaria, including anaemia.[3] The latter relies on artemisinin-based combination therapies (ACTs) which are well tolerated, safe and efficacious.[4] Their use is currently limited to the second and third trimester although the risk of miscarriage, stillbirths or major birth defects when used during the first trimester is better to quinine, the recommended drug.[5,6]

Currently available ACTs for the treatment of Plasmodium falciparum malaria during the second and third trimesters of pregnancy, namely artemether-lumefantrine (AL), artesunate-amodiaquine (ASAQ), mefloquine-artesunate (MAS) and dihydroartemisinin-piperaquine (DP), are generally safe and efficacious.[4] Nevertheless, when considering efficacy and tolerability, DP is probably the best option as it provides a long post-treatment prophylaxis and is well tolerated. DP is also considered for IPTp, as a possible alternative to SP where resistance to this treatment is high [7].[7] Its deployment as IPTp would significantly limit its use as curative treatment. There is the need for increasing the therapeutic options to treat malaria during the 2nd and 3rd trimesters of pregnancy. Pyronaridine-artesunate (PA) may be a possible candidate. PA tablets and granules (Pyramax®) have received a positive European Medicines Agency /Article 58 scientific opinion for the treatment of acute uncomplicated *P. falciparum* or *P. vivax* malaria.[8] The treatment (tablets) is currently registered in 19 African countries and 5 Asian countries. Pyramax (granules) is registered in 15 African countries.

To address this need, we designed a randomized open-label clinical trial to determine the efficacy, and safety of PA versus AL or DP when administered to pregnant women with confirmed *P. falciparum* infection in the second or third trimester.

The specific trial objectives are:

A. To compare the efficacy of PA versus AL or DP in terms of: (i) treatment success (see definition below) 28, 42 and 63 days after the start of treatment, with and without genotyping; (ii) parasite clearance time, including sub-microscopic malaria infections; (iii) gametocyte carriage and clearance; (iv) haematological recovery by Day 7, 14, 28,

For peer review only - http://bmjopen.bmj.com/site/about/guidelines.xhtml

42 and 63 after treatment and at delivery; (v) birth weight measured within 72 hours of delivery and (vi) placental *P. falciparum* malaria.

B. To describe the safety profile of PA, AL and DP in terms of: (i) tolerability and (ii) adverse events (AEs), including serious adverse events (SAEs) and adverse events of special interest (AESI), during the 63-day post-treatment follow-up (mother), at delivery (mother and baby), one month (mother and baby) and one year after the end of pregnancy (baby).

C. To explore the pharmacokinetics of pyronaridine in HIV-infected and non-HIV-infected pregnant women. This will be done on a small number of study subjects (60 women, 30 HIV uninfected and 30 HIV infected), in the 2 countries with the highest HIV prevalence, namely Democratic Republic of the Congo (DRC) and Mozambique.

METHODS AND ANALYSIS

Study setting

The trial will be implemented in five African countries with multiple sites of recruitment as indicated in table 1.

Table 1 Countries and Study site characteristics

Burkina-Faso: Nanoro	The study site is situated in central-west of the country, 90 Km from Ouagadougou in the Nanoro health district catchment area. Malaria transmission is high and seasonal. *P. falciparum* is the most common species of malaria parasite. *Anopheles gambiae* is the main vector transmitting the disease.
Democratic Republic of the Congo: Lisungi	The trial will be carried out at Lisungi health centre, located in Kinshasa suburb, where malaria transmission is perennial. *P. falciparum* is the most common species of malaria parasite. *Anopheles funestus* and *Anopheles gambiae* are the main vector transmitting the disease.
Mali: San and Téné	San health centre is located at 440 km of northeast of Bamako. Téné is at about 50 km south of San. Malaria transmission is high and seasonal. *P. falciparum* is the most common species of malaria parasite. *Anopheles gambiae* is the main vector transmitting the disease.

For peer review only - http://bmjopen.bmj.com/site/about/guidelines.xhtml

Paper draft Version 11
April 12th, 2023

Mozambique: Manhiça District	Manhiça is located in southern Mozambique, 80 Km north of Maputo city. Malaria is endemic with perennial transmission. *P. falciparum* is the most common species of malaria parasite.
The Gambia: Basse, Demba Kunta Koto, Fatoto, Gambissara and Koina	The trial will be carried out in the Upper River Region (eastern part of the country), where the MRCG has a field station. There are 5 health facilities (Basse, Demba Kunta Koto, Fatoto, Gambissara and Koina) from which pregnant women can be recruited. Malaria transmission is seasonal. *P. falciparum* is the most common species of malaria parasite. *Anopheles gambiae* is the main vector transmitting the disease. *Anopheles arabiensis* and *Anopheles funestus* are the main vector transmitting the disease.

The different sites were chosen to include African pregnant women who are residents in different malaria transmission facies including the Gambia which is in pre-elimination phase.

Patient and public involvement

Although patients were not involved in the design of the study, this project was designed to respond to their main concern which is the reduction of malaria adverse effect in pregnant population. The government of the five African countries were engaged through their National Malaria Control Programme which objective is to provide the evidence base to make an additional ACT available for malaria treatment in pregnancy. In case of a positive result, it will be important to integrate this new treatment in (inter) national guidelines. This will be obtained by disseminating as rapidly and efficiently as possible the trial's results to relevant stakeholders, e.g. WHO, National Malaria Control Programmes (NMCPs), and the scientific community working on malaria treatment. Through meetings in all the involved countries, permission from community, religious leaders, and women representatives has been sought before the trial start. Therefore, similar channels will be used to share the study results. Participation in our trial can be burdensome as participants may endure psychological, cost, and physical impacts. However, the project provided special attention such as closer supervision and treatment of any illness to enrolled patients. Also, transport cost has been provided to participants living far from the recruitment centre. Nonetheless, it has been suggested to all participants to withdraw their consent any time if they fell burdened by the study procedures.

For peer review only - http://bmjopen.bmj.com/site/about/guidelines.xhtml

Study design

This is an open-label, multicentre, randomized, non-inferiority clinical trial comparing PA with AL and DP for the treatment of *P. falciparum* malaria in women in the second and third trimesters of pregnancy. The non-inferiority design was suggested based on the hypothesis that PA, which is a newly registered ACT is not worse than the other ACT in terms of safety (for AL) and efficacy (for DP). The rationale for 2 control groups is that AL is currently the most used treatment for malaria in pregnancy while DP seems to be the ACT with the best tolerability and efficacy profile.

The primary hypothesis is the pair-wise non-inferiority of PA as compared to either AL or DP, defined as a difference in the Day 42 PCR-adjusted adequate clinical and parasitological response (ACPR) of <5% (non-inferiority margin).

Participants and procedures

To be included, patients should satisfy all the inclusion criteria while none of the exclusion criteria should be present.

Inclusion criteria

1. Gestation ≥16 weeks and <37 weeks as assessed by ultrasound when possible. If not, height of the uterus or delay of menstruation will be used.
2. *P. falciparum* mono-infection (by microscopy) of any density, regardless of symptoms and HIV status.
3. Haemoglobin ≥7g/dL.
4. Age ≥15 years.
5. Residence within the health facility catchment area.
6. Willingness to adhere to study requirements and to deliver at the health facility.
7. Ability to provide written informed consent; if the woman is minor of age/not emancipated, the consent must be given by a parent or legal guardian according to national law (however, in this case, assent will be obtained to ensure that the woman herself is also freely willing to take part in the study).
8. For the PK study, HIV-infected women should be on first line anti-retroviral treatment for at least 6 months.

Exclusion criteria

For peer review only - http://bmjopen.bmj.com/site/about/guidelines.xhtml

1. Known allergy to the study drugs.
2. History of known pregnancy complications or poor obstetric history such as repeated stillbirths or eclampsia.
3. History or presence of major illnesses likely to influence pregnancy outcome.
4. Any significant illness at the time of screening requiring hospitalization, including:
   i. Severe malaria;
   ii. Any sign or symptom suggesting hepatic lesions (e.g. nausea with abdominal pain and icterus) or severe liver disease classified as B or C by the Child-Pugh score;
   iii. Known history or evidence of clinically significant cardiovascular disorders or family history of long QT syndrome.
5. Intent to move out of the study catchment area before delivery or delivery out of the catchment area.
6. Prior enrolment in the study.
7. Clear evidence of recent (1 week) treatment with antimicrobials with antimalarial activity (azithromycin, clindamycin, tetracycline, quinolones, cotrimoxazole and SP). For HIV-infected pregnant women to be included in the PK sub-study, cotrimoxazole use is not an exclusion criterion.
8. Twin/multiple pregnancy.
9. Known history of G6PD deficiency or sickle cell disease.

Randomisation

Sequence generation

Women recruited in the trial will be randomly allocated to one of the three treatment arms of the study according to a randomization list generated using R software prior any study activity. The block randomisation technique will be used to achieve balance in the allocation of participants to treatment arms. A varying size of the blocks will be setup to reduce selection bias by using random block sizes and keeping the investigator blind to the size of each block, particularly in this open randomized control arm study. The randomisation will be done per study site within each country and according to the specific sample size allocated to each site. The data management team in each country, in coordination with the study statistician and the study Data management Centre, will print the randomization code containing the study arm and put into a sealed envelope numbered sequentially and containing the treatment arm to which the patient should be allocated.

For peer review only - http://bmjopen.bmj.com/site/about/guidelines.xhtml

For peer review only

Paper draft Version 11
April 12th, 2023

Recruitment

Women in the 2nd or 3rd trimester of pregnancy will be screened for malaria with a Rapid Diagnostic Test based on the detection of Histidine-Rich Protein 2 (HRP2) and Parasite lactate; positive results will be confirmed by microscopy. Women with a confirmed *P. falciparum* infection will be asked to provide a written informed consent covering all trial procedures and be assigned a screening number. All women meeting the entry criteria will be given a randomization number. Data of screened and randomized women will be kept in a logbook.

Investigational product and comparators

Pyronaridine-artesunate: Pyramax® (Shin Poong Pharmaceutical Company, Korea)

PA, is a film-coated tablet containing 180 mg pyronaridine tetraphosphate and 60 mg artesunate. The tablets should be taken orally once daily for three days and according to body weight as follows 24-<45Kg 2 tablets; 45-<65Kg 3 tablets; ≥65Kg 4 tablets. This dosage regimen provides a daily dose of 7.2-13.8 mg/kg pyronaridine and 2.4-4.6 mg/kg artesunate. PA can be administered at any time, regardless of food consumption.

Dihydroartemisinin-piperaquine (Alfasigma, Italy)

DP is a white film-coated tablet composed of 40 mg dihydroartemisinin and 320 mg piperaquine. DP tablets should be taken orally once daily for three days as follows 24-<36Kg 2 tablets; 36-<75Kg 3 tablets; ≥75Kg 4 tablets.[9],[10]

Artemether-lumefantrine: Coartem® (Novartis)

AL tablets contain 80 mg artemether and 480 mg lumefantrine. This is a fixed-dose combination of artemether (a semi synthetic artemisinin derivative) and lumefantrine (a slowly eliminated drug also referred to as benflumetol).[11] AL 80 mg/480 mg tablets should be taken orally twice daily for three days as follows to patients weighing 35 kg and above:

- 1st dose, at the time of initial diagnosis (Day 0): 1 tablet
- 2nd dose, at 8 hours after the 1st dose: 1 tablet
- 3rd dose, in the morning of the Day 1: 1 tablet
- 4th dose, in the evening of Day 1: 1 tablet
- 5th dose, in the morning of Day 2: 1 tablet
- 6th dose, in the evening of Day 2: 1 tablet

For peer review only - http://bmjopen.bmj.com/site/about/guidelines.xhtml

Paper draft Version 11
April 12th, 2023

Explanation for the choice of comparators.

Pregnant women (2nd or 3rd trimester) will be recruited for the study and allocated to one of the three treatment groups. The choice of two control arms (AL and DP) is justified by AL being the most commonly used treatment for malaria in African pregnant women, while DP has several advantages compared to AL, namely higher efficacy and longer post-treatment prophylaxis.

Study outcomes

Primary endpoint

The primary endpoint is treatment efficacy determined as the proportion of women with PCR-adjusted ACPR at Day 42, i.e. all women not having met the criteria of treatment failure (Supplementary Table 1). Recurrent infections classified by genotyping as new infection will not be considered as treatment failure.

Secondary endpoints

Safety is the main secondary endpoint and includes adverse events detected during active follow-up (63 days post-treatment), including significant changes in relevant laboratory values, those detected at delivery, at 4-6 weeks and 1 year after birth.

Others secondary endpoints include:

1. PCR-adjusted ACPR at Days 28 and 63;
2. PCR unadjusted ACPR on Days 28, 42 and 63;
3. Parasite and fever elimination time;
4. Gametocyte carriage and clearance;
5. Hematologic recovery, i.e., Hb changes between Day 0 and Days 7, 14, 28, 42, 63 and at delivery;
6. Placenta malaria (recent, past, and chronic infection);
7. Average BW and prevalence of low birth weight (<2,500g).

Exploratory endpoints

We will explore: (i) the drug exposure and key pharmacokinetic parameters of pyronaridine in HIV uninfected pregnant women and (ii) the drug exposure and key pharmacokinetic parameters of HIV-infected pregnant women on antiretroviral therapy. Drug exposure is defined as the area under the whole blood concentration versus time curve from zero to infinity,

For peer review only - http://bmjopen.bmj.com/site/about/guidelines.xhtml

For peer review only

AUC $_{0-\infty}$. The main pharmacokinetic parameters that will be evaluated are absorption rate (k_a), drug clearance (CL/F) and volume of distribution (V_d/F).

Sample size

The sample size was estimated assuming an efficacy for PA (PCR adjusted at Day 42) of at least 95% and a non-inferiority margin of 5%; non-inferiority will be tested using raw pooling of country data, using a Wilson's interval of proportion difference. The lower limit of the Wilson score interval of 97.5% of the AL (or DP) proportion of ACPR - the PA proportion of ACPR must be greater than -5% to claim non-inferiority. In addition, an adjusted non-inferiority analysis will allow for country effect[12]. Assuming 20% of loss to follow-up, the total sample size for 90% power would be 3*500/0.8 = 1875, or 375 pregnant women per country and 125 per arm per country. The sample size calculated will maintain the power of the adjusted stratified non-inferiority test.

Study implementation and timeline

Participants' assignment into study arm is under the responsibility of study qualified physicians. All physician participating in PYRAPREG study have been trained in Good Clinical Practices and all study requirements.

Pregnant women fulfilling the inclusion/exclusion criteria will be recruited during antenatal clinics over a period of about 30 months. Scheduled visits will be at Day 3, 7 and then every week until Day 63 post-treatment. A window period is allowed if study subjects are unable to attend on the scheduled date, i.e. ± 1-day for Day 7 and 14; ± 2 for Day 21 and 28; and ± 3 from Day 35 to Day 63. Women will be encouraged to attend the antenatal clinic between scheduled visits if sick.

Pregnant women recruited during the third trimester (before 37 weeks) may deliver before the end of the 63-day active follow up. In this case, the assessment at delivery will be done as planned but the active follow up will continue after delivery until Day 63. A blood sample to measure liver function test (LFT) and bilirubin will be taken within 48 hours of delivery from babies whose mothers delivered within 2 weeks of the inclusion in the trial.

The outcome of pregnancy, birth weight and maternal Hb will be collected as soon as possible after delivery. A placenta biopsy will be collected for later histopathological analysis. The new-born will be examined for congenital abnormalities. Both the mother and the new-born will be

For peer review only - http://bmjopen.bmj.com/site/about/guidelines.xhtml

reassessed for any adverse event between 4 and 6 weeks and then after one year (only the baby). Patients will be assessed as summarized in the study visit schedule (Supplementary Table 2).

Data collection and management

Plans for assessment and collection of outcomes

Study visits: At each visit, both scheduled and unscheduled, the medical history since the last visit (including any treatment taken), current signs and symptoms (if any) will be collected. A blood sample for thick smear will be collected and the body temperature checked. Dried blood spots for later genotyping to distinguish between recrudescence and new infection will be collected at Day 0, before treatment, and at every study visit. Information on any adverse event will also be collected. Haematology (full blood count) will be performed at Days 0, 7, 14, 28, 42 and 63; biochemistry (Total and conjugated bilirubin, AST, ALT, alkaline phosphatase, and creatinine) at Day 0, 1, and 7. In the event of increased liver function tests (LFTs) >3xULN, the result will be verified by taking an additional sample to be analysed (within 24 hours). Subsequent blood samples for LFTs will be taken at 48-hour intervals until the results return to <2xULN. ECG will be performed at Day 0, before drug intake, and at Day 2 after drug intake. If abnormal at Day 2, ECG will be repeated at Day 7 and every week until return to normal. At the end of the active follow up, on Day 63, field assistants will visit the study subjects monthly to maintain the contact, but without collecting any data or biological samples.

Babies born from women who deliver within or 2 weeks after the active follow up will have a blood sample taken to measure LFT and bilirubin within 48 hours of delivery.

The outcome of pregnancy, birth weight and maternal Hb will be collected as soon as possible after delivery. A placenta biopsy will be collected, put immediately in 10% buffered formalin container stored in 4°C at the study site for later histopathological analysis. The new-born will be examined for congenital abnormalities. Both the mother and the new-born will be reassessed twice after delivery for any adverse event: between 4 and 6 weeks and then after one year.

PCR analysis

Genotyping of recurrent infections will be performed by characterizing the merozoites surface protein 1 (*msp1*), *msp2* and glutamate rich protein (*glurp)* genes, single-copy genes of the *P. falciparum* genome. PCR amplification of deoxyribonucleic acid (DNA) from a single parasite clone will yield a unique amplification product. For all three genes, each PCR amplification product of different size is considered from a different *P. falciparum* clone and reflects a different genotype. For samples collected from the same patient on Day 0 and on the Day of

For peer review only - http://bmjopen.bmj.com/site/about/guidelines.xhtml

Paper draft Version 11
April 12th, 2023

recurrent parasitaemia (after Day 3), the length polymorphism of *msp1*, *msp2* and *glurp* will be determined, i.e., the number of bands in each PCR reaction and their respective sizes. The results will be interpreted as follows:

Recurrence: at least one polymorphism of identical length for each marker (*msp1*, *msp2* and *glurp*) is found in the sample collected on Day 0 and on the Day of the recurrent parasitaemia.

New infection: For at least one marker, the length polymorphism is different between the sample collected on Day 0 and on the Day of recurrent parasitaemia.

Indeterminate: Samples that did not give a result due to an inability to amplify DNA on Day 0 and/or on the Day of recurrent parasitaemia.

Analysis of placental samples

The placental biopsy samples will be processed and embedded in paraffin wax using standard .techniques. Paraffin sections 4 µm thick will be stained with haematoxylin-eosin. Placental biopsies after reading will be classified according to the following definitions[13]: (i) acute infection (parasite present, hemozoin absent or minimal deposition); (ii) chronic infection (parasites and heavy hemozoin deposition); (iii) past infection (no parasite but presence of hemozoin) or (iv) no infection (absence of parasites and hemozoin).

Haematological and biochemical analysis

Haematology (including haemoglobin) and biochemistry (including LFTs, i.e., AST, ALT, ALP, total and conjugated bilirubin) will be performed during active follow-up; haematology will be performed prior to the first dose of treatment, on Day 0, and then on Days 7, 14, 28, 42, and 63. An additional test will be performed at any unscheduled visit. In addition, only Hb will be measured at delivery. Biochemistry will also be done on Day 0, prior to treatment, then on Day 1 and Day 7. If LFTs increase more than 3 times the upper limit normal (ULN), the result will be verified by taking another sample for analysis as soon as possible (within 24 hours of the initial sample). Subsequent blood samples for LFTs will be taken at 48-hour intervals until results return to < 2xULN.

Statistical methods

Statistical methods for primary and secondary outcomes

The baseline characteristics will be described by treatment group and site. No statistical tests of significance will be undertaken, although the clinical significance of any imbalance will be noted.

For peer review only - http://bmjopen.bmj.com/site/about/guidelines.xhtml

The primary analysis will be the assessment of the non-inferiority of the PA compared to the DP and AL for the PCR-adjusted ACPR at Day 42. It will use the combined data from the five countries, with adjustment for any centre effect, using an additive model for response rates (i.e., a generalised linear model with a Bernoulli error distribution and an identity link function). This will allow the evaluation of two pairwise treatment comparisons, i.e., PA versus AL, PA versus DP.

Efficacy analysis (primary end point)

For the efficacy analysis, both a m-ITT approach and a PP approach will be adopted, with PP analysis being the main approach, as recommended for equivalence studies. The m-ITT population will include all participants who have received any amount of study drug and have confirmed *P. falciparum* infection prior treatment.

The PP population will consist of all participants meeting the following predefined criteria:

1. Fulfilling the entry criteria specified in the clinical study protocol.
2. Completed treatment, including not having vomited the study drug or, if vomited, received a repeat dose that was not vomited.
3. No previous or concomitant medication that would interfere with treatment outcome.

The PP population will be identified after locking the database, just before the statistical analysis.

For the primary endpoint, i.e. treatment efficacy at Day 42, the proportion of participants with PCR-adjusted ACPR will be determined by treatment arm. Similar procedures will be applied to the m-ITT population.

Safety analysis (secondary end point)

The safety population will include all participants randomised and treated with at least one dose of the three antimalarial treatments. Standard safety report tables summarize and list safety data. All AEs will be coded using the most recent version of the Medical Dictionary for Regulatory activities (MedDRA) dictionary. Treatment emergent AEs will be defined as all AEs which started after the first administration of study drug. These AEs will be summarized by primary system organ class and preferred term, separately for each treatment regimen and overall. Similar summaries will be provided for treatment emergent AEs considered to be related to study treatments. In addition, treatment emergent AEs will be summarized by primary system organ class, preferred term, and maximal severity.

For peer review only - http://bmjopen.bmj.com/site/about/guidelines.xhtml

Vital signs and routine safety laboratory data will be summarised descriptively by treatment regimen and overall, by time point. Absolute values and changes from baseline will be presented. Safety laboratory data will be classified according to the normal ranges (below, within, above) and summaries of changes from baseline in these categories will be provided by treatment regimen and overall. Further, safety laboratory values will be classified according to Common Terminology Criteria for Adverse Events (CTCAE) and shift tables of the baseline CTCAE category versus post baseline categories will be presented.

Pharmacokinetic analysis

PA availability in patients' blood will be compared between pregnant women infected and non-infected by HIV on day 0 before and 1 hour (hr), 2hr, 6hr and 10hr after the first PA dose. Additional comparison will be done on day 1 (24hr after the first PA dose while before the second PA dose), day 2 (before the last PA dose), day 7, 14, 21, 28 and 42. Mean of PA concentration will be estimated and compared between pregnant and non-pregnant women over time. Similar figure will be used to compared HIV infected and non-infected pregnant women.

Study monitoring

The trial will be evaluated by monitors (a Clinical Research Organization will be contracted) (pre-study visit) for its preparedness to carry out, following by regular monitoring and closeout visits.

The coordination of the whole project is in the hand of the sponsor, which will be the primary contact contact. He will be assisted by appropriate administrative and financial staff. A Coordinating Committee (CC) including one member of each institution will be main decision body of the consortium. For the trial, there will be three entities involved in its implementation and management, namely the Data Safety and Monitoring Board (DSMB), the Trial Steering Committee (TSC), and the Trial Management Group (TMG). Both DSMB and TSC are composed of independent experts to provide the overall supervision of the trial, monitor trial progress and advise on scientific credibility.

Ethics and dissemination

This protocol has been approved by Ethics Committee for Health Research (CERS) in Burkina Faso (Reference: 2020-3-047), the National Health Ethics Committee (CNES) in Democratic

For peer review only - http://bmjopen.bmj.com/site/about/guidelines.xhtml

Republic of Congo (Reference: 169/CNES/BN/PMMF/2019), the Ethics Committee of the Faculty of Medicine and Odontostomatology (FMOS) / Faculty of Pharmacy (FPHA) in Mali (Reference: 2020/46/CE/FMOS/FAPH) in Mali, The Gambia Government/MRCG Joint Ethics Committee (Reference: 21818) and the National Bioethics Committee for Health (CNBS) in Mozambique (Reference: 313/CNBS/20).

Written informed consent will be taken from each individual prior to her participation in the study, the study investigators.

The outcomes of the project will be communicated to the National Malaria Control Programme of the respective countries and to the Global Malaria Programme of the World Health Organization. The results will be published in peer reviewed Open Access Journals and presented at (inter)national conferences and meetings.

A work package is focused on the development of a plan for internal and external project communication, on the development of communication tools, and on the dissemination and exploitation of the project's findings. All these activities will be in line with H2020 guidelines on dissemination and publication of results and will highlight the contribution of the EDCTP in tackling societal and health challenges.

Trial status

Active recruitment

Abbreviations

ACPR	Adequate clinical and parasitological response
ACT	Artemisinin-based combination therapy
AE	Adverse event
AESI	Adverse event of particular interest
AL	Artemether-lumefantrine
ALP	Alkaline phosphatase
ALT	Alanine aminotransferase
AMC	Academic Medical Centre
AST	Aspartate aminotransferase
CC	Coordinating Committee
CISM	Centro de Investigação em Saúde da Manhiça

For peer review only - http://bmjopen.bmj.com/site/about/guidelines.xhtml

CRO	Contract Research Organization
CRUN	Clinical Research Unit of Nanoro
CTCAE	Common Terminology Criteria for Adverse Events
DNA	deoxyribonucleic acid
DP	Dihydroartemisinin-piperaquine
DSMB	Data Safety Monitoring Board
eCRF	Electronic case report form
EDCTP	European & Developing Countries Clinical Trials Partnership
ETF	Early treatment failure
FAPH	Faculty of Pharmacy
FMOS	Faculty of Medicine and Odontostomatology
GCP	Good Clinical Practice
glurp	Glutamate rich protein
HIV	Human immunodeficiency virus
HS-RDT	Highly sensitive rapid diagnostic test
IPTp-SP	Intermittent preventive treatment during pregnancy with sulfadoxine-pyrimethamine
IRSS	Institut de Recherche en Sciences de la Santé
MedDRA	Medical Dictionary for Regulatory activities
mITT	Modified Intention-to-treat
LFT	Liver Function Tests
LBW	Low birth weight
LCF	Late Clinical Failure
LPF	Late parasitological failure
LTF	Late treatment failure
MAS	Mefloquine-artesunate
MMV	Medicine of Malaria Venture
MRTC	Malaria Research and Training Center
MRCG	The MRC Unit The Gambia
msp1	Merozoites Surface Protein gene 1
NKI	Nederlands Kanker Institutu
NOAEL	No observed adverse effect level
P. falciparum	*Plasmodium falciparum*

For peer review only - http://bmjopen.bmj.com/site/about/guidelines.xhtml

Paper draft Version 11
April 12th, 2023

P. vivax	*Plasmodium vivax*
PA	Pyronaridine-artesunate
PCR	Polymerase chain reaction
PP	Per protocol analysis
REDCap	Research Electronic Data Capture
SAE	Serious adverse event
SP	Sulfadoxine-pyrimethamine
TMG	Trial management group
TSC	Trial Steering Committee
ULN	Upper Limit Normal
UNIKIN	University of Kinshasa
USTTB	University of Sciences of Techniques and Technologies of Bamako
WANECAM	West African Network for Antimalarial Drugs
WHO	World Health Organization

Acknowledgments

The consortium thanks Shinpoong Pharm.co., LTd for providing the study drug (pyronaridine-artesunate: Pyramax) and the Medicine of Malaria Venture (MMV) for technical support and assistance to this ongoing clinical trial. The project would like to thank patients and their advisors for their contribution to the study implementation.

Authors' information

Moussa Djimde: mdjimde@icermali.org
Japhet Kabalu Tshiongo: kabalujaphet@gmail.com
Hypolite Muhindo-Mavoko: hypomavoko@gmail.com
Halidou Tinto: halidoutinto@gmail.com
Esperanca Sevene: esevene68@gmail.com
Maminata Traoré: traoré_maminata@yahoo.fr
Anifa Vala: anifa.vala@manhica.net
Salesio Macuacua: salesio.macuacua@manhica.net
Bérenger Kaboré: kaboreberenger@gmail.com
Edgard Diniba Dabira: edabira@mrc.gm
Annette Erhart: Annette.Erhart@lshtm.ac.uk

For peer review only - http://bmjopen.bmj.com/site/about/guidelines.xhtml

Paper draft Version 11
April 12th, 2023

Hamadoun Diakite: diakiteh@icermali.org

Mohamed Keita: medkeita@gmail.com

Mireia Piqueras: mireia.piqueras@isglobal.org

Raquel González: Raquel.gonzalez@isglobal.org

Clara Menéndez: Clara.menendez@isglobal.org

Thomas P. C. Dorlo: t.dorlo@nki.nl

Issaka Sagara: isagara@icermali.org

Petra Mens: p.f.mens@amsterdamumc.nl

Henk Schallig: h.d.schallig@amsterdamumc.nl

Umberto D'Alessandro: udalessandro@mrc.gm

Kassoum Kayentao: kayentao@icermali.org

Author's contributions

The following co-authors were responsible for the conception and design of the clinical trial : KK-Kassoum Kayentao, UDA-Umberto D'Alessandro, HMM-Hypolite Muhindo-Mavoko, HT-Halidou Tinto, MT-Maminata Traore, ES-Esperanca Sevene, MP-Mireia Piqueras, RG-Raquel Gonzalez, CM-Clara Menendez, TPCD-Thomas P.C. Dorlo, PFM-Petra F. Mens, HDFHS-Henk D. Schallig. The first draft of the paper was written by MD-Moussa Djimde and JKT-Japhet Kabalu Tshiongo. IS-Issaka Sagara is responsible for the statistical analysis. All authors and the following (AV-Anifa Vala, SM-Salesio Macuacua, BK-Berenger Kabore, EDD-Edgard Diniba Dabira, AE-Annette Erhart, HD-Hamadoun Diakite, MK-Mohamed Keita) contributed to critical review and approved the final manuscript.

Competing interests

None of the authors declare a conflict of interests.

Funding statement

This project is part of the EDCTP2 programme supported by the European Union (grant number RIA2017MC-2025-PYRAPREG).

REFRENCES

For peer review only - http://bmjopen.bmj.com/site/about/guidelines.xhtml

1 Takem EN, D'Alessandro U. MALARIA IN PREGNANCY. *Mediterr J Hematol Infect Dis* 2013;**5**:e2013010. doi:10.4084/mjhid.2013.010

2 Desai M, ter Kuile FO, Nosten F, *et al.* Epidemiology and burden of malaria in pregnancy. Lancet Infectious Diseases. 2007. doi:10.1016/S1473-3099(07)70021-X

3 Rogerson SJ, Desai M, Mayor A, *et al.* Burden, pathology, and costs of malaria in pregnancy: new developments for an old problem. The Lancet Infectious Diseases. 2018. doi:10.1016/S1473-3099(18)30066-5

4 D'Alessandro U, Hill J, Tarning J, *et al.* Treatment of uncomplicated and severe malaria during pregnancy. The Lancet Infectious Diseases. 2018. doi:10.1016/S1473-3099(18)30065-3

5 Dellicour S, Sevene E, McGready R, *et al.* First-trimester artemisinin derivatives and quinine treatments and the risk of adverse pregnancy outcomes in Africa and Asia: A meta-analysis of observational studies. *PLoS Medicine* Published Online First: 2017. doi:10.1371/journal.pmed.1002290

6 Marrelli MT, Brotto M, Sharma H, *et al.* The effect of malaria and anti-malarial drugs on skeletal and cardiac muscles. *Malaria Journal* 2016;**15**:524. doi:10.1186/s12936-016-1577-y

7 Meghna Desai, Jenny Hill, Silke Fernandes, Patrick Walker, Christopher Pell, Julie Gutman, Kassoum Kayentao, Raquel Gonzalez, Jayne Webster, Brian Greenwood, Michel Cot FOTK. Prevention of malaria in pregnancy. *Lancet Infect Dis* 2018;**18(4):e119**.

8 European Public Assessment Report (EPAR). Assessment report; Pyramax. https://www.ema.europa.eu/en/documents/medicine-outside-eu/pyramax-public-assessment-report_en.pdf; Accessed on 17 February 2021. 2016.

9 D'Alessandro U. Existing antimalarial agents and malaria-treatment strategies. Expert Opinion on Pharmacotherapy. 2009. doi:10.1517/14656560902942319

10 Artemisinin-Based Treatments in Pregnant Women with Malaria. *New England Journal of Medicine* Published Online First: 2016. doi:10.1056/nejmc1604709

11 Muhindo Mavoko H, Nabasumba C, Tinto H, *et al.* Impact of retreatment with an artemisinin-based combination on malaria incidence and its potential selection of resistant strains: study protocol for a randomized controlled clinical trial. *Trials 2013 14:1* 2013;**14**:1–15. doi:10.1186/1745-6215-14-307

12 Yan X, Su XG. Stratified Wilson and Newcombe Confidence Intervals for Multiple Binomial Proportions. *Statistics in Biopharmaceutical Research* Published Online First: 2010. doi:10.1198/sbr.2009.0049

13 Ismail MR, Ordi J, Menendez C, *et al.* Placental pathology in malaria: A histological, immunohistochemical, and quantitative study. *Human Pathology* Published Online First: 2000. doi:10.1016/S0046-8177(00)80203-8

For peer review only - http://bmjopen.bmj.com/site/about/guidelines.xhtml

Paper draft Version 11
April 12th, 2023

For peer review only

For peer review only - http://bmjopen.bmj.com/site/about/guidelines.xhtml

Table legend

Table 1: Definition of early and late treatment failures

Table 2: Study visit schedule

Table 1: Definition of early and late treatment failures

**Early treatment failure (ETF)** one of the following	**Late treatment failure (LTF)**
1) Development of danger signs or severe malaria on Days 0-3 with parasitaemia<br><br>2) Presence of parasitaemia on Day 3 with fever (axillary temperature ≥ 37.5°C)	Late Clinical Failure (LCF):<br>1) Development of danger signs or severe malaria on any day after Day 3 in the presence of parasitaemia, without having previously met any of the ETF criteria;<br>2) Presence of parasitaemia and fever on any day after Day 3, without having previously met the ETF criteria.<br><br>Late parasitological failure (LPF):<br>Presence of parasitaemia on any day, starting on Day 7, and axillary temperature <37.5 ºC, without previously meeting any of the ETF or LCF criteria.

For peer review only

For peer review only

Table 2: Study visit schedule

Day	0	1	2	3	7(± 1)	14 (± 1)	21 (± 2)	28 (± 2)	35 (± 3)	42 (± 3)	49-56 (± 3)	63 (± 3)	Any other day[1]	Delivery	4-6 weeks post-end of pregnancy	1-year post-end of pregnancy
History (symptoms)	X	X	X	X	X	X	X	X	X	X	X	X	X			
Informed consent	X															
Examination (clinical)	X	X	X	X	X	X	X	X	X	X	X	X	X	X		
Vital signs	X	X	X	X	X	X	X	X	X	X	X	X	X	X		
Foetal viability	X			X	X	X	X	X	X	X	X	X	X			
Blood Pressure	X	X	X	X	X	X	X	X	X	X	X	X	X	X		
Body Temperature	X	X	X	X	X	X	X	X	X	X	X	X	X	X		
ECG	X		X		X[2]											
Blood slide[3]	X	X	X	X	X	X	X	X	X	X	X	X	X	X		
Filter paper for genotyping	X	X	X	X	X	X	X	X	X	X	X	X	X	X		
Adverse drug reactions	X	X	X	X	X	X	X	X	X	X	X	X	X	X	X	
Concomitant medications	X	X	X	X	X	X	X	X	X	X	X	X	X	X	X	
Haematology	X				X	X		X		X		X	X	X[6]		
Treatment administration	X	X	X													
Blood sample for PK[4]	X	X	X		X	X	X	X		X						
Biochemistry[5]	X	X			X											
Urine analysis	X							X		X		X				
Placenta biopsy														X		
Newborn Assessment														X[8]	X	
Newborn LFT														X[7]		
Infant assessment																X

[1]Spontaneous attendance to the health facility (unscheduled visit); [2]ECG at Day 7 if abnormal at Day 2; [3]Thick and thin blood film; [4]Four blood samples at scheduled times, first sampling before treatment administration;[5] In the event of increased LFTs >3xULN, the result will be verified by taking a further sample for analysis as soon as possible (within 24 hours of the original sample). Subsequent blood samples for LFTs will be taken at 48-hour intervals until the results return to $\leq$2xULN;[6] Only Hb; [7]If delivery within or 2 weeks after the active follow up; [8]Blood sample collected systematically for dried blood spots, blood slide and Hb.

SPIRIT

STANDARD PROTOCOL ITEMS: RECOMMENDATIONS FOR INTERVENTIONAL TRIALS

SPIRIT 2013 Checklist: Recommended items to address in a clinical trial protocol and related documents*

Section/item	Item No	Description	Addressed on page number
**Administrative information**			
Title	1	Descriptive title identifying the study design, population, interventions, and, if applicable, trial acronym	________1______
Trial registration	2a	Trial identifier and registry name. If not yet registered, name of intended registry	________2______
	2b	All items from the World Health Organization Trial Registration Data Set	______________
Protocol version	3	Date and version identifier	_______2________
Funding	4	Sources and types of financial, material, and other support	______ 18-19______
Roles and responsibilities	5a	Names, affiliations, and roles of protocol contributors	______18_________
	5b	Name and contact information for the trial sponsor	______2_________
	5c	Role of study sponsor and funders, if any, in study design; collection, management, analysis, and interpretation of data; writing of the report; and the decision to submit the report for publication, including whether they will have ultimate authority over any of these activities	______2_________
	5d	Composition, roles, and responsibilities of the coordinating centre, steering committee, endpoint adjudication committee, data management team, and other individuals or groups overseeing the trial, if applicable (see Item 21a for data monitoring committee)	______2_________

For peer review only

Introduction

Background and rationale	6a	Description of research question and justification for undertaking the trial, including summary of relevant studies (published and unpublished) examining benefits and harms for each intervention	__5_________
	6b	Explanation for choice of comparators	___5, 9-10______
Objectives	7	Specific objectives or hypotheses	____5,6_______
Trial design	8	Description of trial design including type of trial (eg, parallel group, crossover, factorial, single group), allocation ratio, and framework (eg, superiority, equivalence, noninferiority, exploratory)	_____5,7_______

Methods: Participants, interventions, and outcomes

Study setting	9	Description of study settings (eg, community clinic, academic hospital) and list of countries where data will be collected. Reference to where list of study sites can be obtained	_____8________
Eligibility criteria	10	Inclusion and exclusion criteria for participants. If applicable, eligibility criteria for study centres and individuals who will perform the interventions (eg, surgeons, psychotherapists)	____8-9________
Interventions	11a	Interventions for each group with sufficient detail to allow replication, including how and when they will be administered	__9-10________
	11b	Criteria for discontinuing or modifying allocated interventions for a given trial participant (eg, drug dose change in response to harms, participant request, or improving/worsening disease)	____9-10_______
	11c	Strategies to improve adherence to intervention protocols, and any procedures for monitoring adherence (eg, drug tablet return, laboratory tests)	____8-9,12-13___
	11d	Relevant concomitant care and interventions that are permitted or prohibited during the trial	____9_________
Outcomes	12	Primary, secondary, and other outcomes, including the specific measurement variable (eg, systolic blood pressure), analysis metric (eg, change from baseline, final value, time to event), method of aggregation (eg, median, proportion), and time point for each outcome. Explanation of the clinical relevance of chosen efficacy and harm outcomes is strongly recommended	__10-11_______
Participant timeline	13	Time schedule of enrolment, interventions (including any run-ins and washouts), assessments, and visits for participants. A schematic diagram is highly recommended (see Figure)	_____12, 25__

For peer review only - http://bmjopen.bmj.com/site/about/guidelines.xhtml

Sample size	14	Estimated number of participants needed to achieve study objectives and how it was determined, including clinical and statistical assumptions supporting any sample size calculations	_11____________
Recruitment	15	Strategies for achieving adequate participant enrolment to reach target sample size	__9, 12________
**Methods: Assignment of interventions (for controlled trials)**			
Allocation:			
Sequence generation	16a	Method of generating the allocation sequence (eg, computer-generated random numbers), and list of any factors for stratification. To reduce predictability of a random sequence, details of any planned restriction (eg, blocking) should be provided in a separate document that is unavailable to those who enrol participants or assign interventions	___9__________
Allocation concealment mechanism	16b	Mechanism of implementing the allocation sequence (eg, central telephone; sequentially numbered, opaque, sealed envelopes), describing any steps to conceal the sequence until interventions are assigned	______9_______
Implementation	16c	Who will generate the allocation sequence, who will enrol participants, and who will assign participants to interventions	_______9______
Blinding (masking)	17a	Who will be blinded after assignment to interventions (eg, trial participants, care providers, outcome assessors, data analysts), and how	________9_____
	17b	If blinded, circumstances under which unblinding is permissible, and procedure for revealing a participant's allocated intervention during the trial	______8_______
**Methods: Data collection, management, and analysis**			
Data collection methods	18a	Plans for assessment and collection of outcome, baseline, and other trial data, including any related processes to promote data quality (eg, duplicate measurements, training of assessors) and a description of study instruments (eg, questionnaires, laboratory tests) along with their reliability and validity, if known. Reference to where data collection forms can be found, if not in the protocol	_____12-13_____
	18b	Plans to promote participant retention and complete follow-up, including list of any outcome data to be collected for participants who discontinue or deviate from intervention protocols	______12-13___

Data management	19	Plans for data entry, coding, security, and storage, including any related processes to promote data quality (eg, double data entry; range checks for data values). Reference to where details of data management procedures can be found, if not in the protocol	_______12-13____
Statistical methods	20a	Statistical methods for analysing primary and secondary outcomes. Reference to where other details of the statistical analysis plan can be found, if not in the protocol	_____14-15______
	20b	Methods for any additional analyses (eg, subgroup and adjusted analyses)	___14-15_______
	20c	Definition of analysis population relating to protocol non-adherence (eg, as randomised analysis), and any statistical methods to handle missing data (eg, multiple imputation)	_____15_________
**Methods: Monitoring**			
Data monitoring	21a	Composition of data monitoring committee (DMC); summary of its role and reporting structure; statement of whether it is independent from the sponsor and competing interests; and reference to where further details about its charter can be found, if not in the protocol. Alternatively, an explanation of why a DMC is not needed	_____16_________
	21b	Description of any interim analyses and stopping guidelines, including who will have access to these interim results and make the final decision to terminate the trial	_____14_____
Harms	22	Plans for collecting, assessing, reporting, and managing solicited and spontaneously reported adverse events and other unintended effects of trial interventions or trial conduct	___14______
Auditing	23	Frequency and procedures for auditing trial conduct, if any, and whether the process will be independent from investigators and the sponsor	_____16_________
**Ethics and dissemination**			
Research ethics approval	24	Plans for seeking research ethics committee/institutional review board (REC/IRB) approval	______16________
Protocol amendments	25	Plans for communicating important protocol modifications (eg, changes to eligibility criteria, outcomes, analyses) to relevant parties (eg, investigators, REC/IRBs, trial participants, trial registries, journals, regulators)	__16___________

Consent or assent	26a	Who will obtain informed consent or assent from potential trial participants or authorised surrogates, and how (see Item 32)	___16__________
	26b	Additional consent provisions for collection and use of participant data and biological specimens in ancillary studies, if applicable	____NA________ _
Confidentiality	27	How personal information about potential and enrolled participants will be collected, shared, and maintained in order to protect confidentiality before, during, and after the trial	____16_________
Declaration of interests	28	Financial and other competing interests for principal investigators for the overall trial and each study site	_____20_________
Access to data	29	Statement of who will have access to the final trial dataset, and disclosure of contractual agreements that limit such access for investigators	___9.13. 17____
Ancillary and post-trial care	30	Provisions, if any, for ancillary and post-trial care, and for compensation to those who suffer harm from trial participation	_______NA____
Dissemination policy	31a	Plans for investigators and sponsor to communicate trial results to participants, healthcare professionals, the public, and other relevant groups (eg, via publication, reporting in results databases, or other data sharing arrangements), including any publication restrictions	___16__________
	31b	Authorship eligibility guidelines and any intended use of professional writers	_____None_____
	31c	Plans, if any, for granting public access to the full protocol, participant-level dataset, and statistical code	_____None_____
**Appendices**			
Informed consent materials	32	Model consent form and other related documentation given to participants and authorised surrogates	_____26-40____
Biological specimens	33	Plans for collection, laboratory evaluation, and storage of biological specimens for genetic or molecular analysis in the current trial and for future use in ancillary studies, if applicable	_____12-14____

*It is strongly recommended that this checklist be read in conjunction with the SPIRIT 2013 Explanation & Elaboration for important clarification on the items. Amendments to the protocol should be tracked and dated. The SPIRIT checklist is copyrighted by the SPIRIT Group under the Creative Commons "Attribution-NonCommercial-NoDerivs 3.0 Unported" license.

For peer review only - http://bmjopen.bmj.com/site/about/guidelines.xhtml

BMJ Open

Efficacy and safety of pyronaridine-artesunate (PYRAMAX□) for the treatment of P. falciparum uncomplicated malaria in African pregnant women (PYRAPREG): study protocol for a phase 3, non-inferiority, randomized open-label clinical trial

Journal:	*BMJ Open*
Manuscript ID	bmjopen-2022-065295.R2
Article Type:	Protocol
Date Submitted by the Author:	30-Aug-2023

Complete List of Authors:	Djimde, Moussa; University of Sciences Techniques and Technologies of Bamako, Malaria Research and Training Center<br>Tshiongo, Japhet ; Universite de Kinshasa, Département of Tropical Médecine<br>Muhindo, Hypolite ; Universite de Kinshasa Faculte de Medecine, Department of Tropical Medicine<br>Tinto, Halidou; Institut de Recherche en Sciences de la Santé (IRSS) – Unité de Recherche Clinique de Nanoro, Burkina Faso Ouagadougou, BF<br>Sevene, Esperanca; Centro de Investigacao em Saude de Manhica; Universidade Eduardo Mondlane<br>Traore, Maminata; Institut de Recherche en Sciences de la Santé (IRSS) – Unité de Recherche Clinique de Nanoro, Burkina Faso<br>Vala, Anifa; Centro de Investigação em Saúde de Manhiça<br>Macuacua, Salesio; Centro de Investigação em Saúde da Manhiça (CISM), Mozambique<br>Kabore, Berenger; Institut de Recherche en Sciences de la Santé (IRSS) – Unité de Recherche Clinique de Nanoro, Burkina Faso<br>Dabira, Edgard; MRC Unit The Gambia (MRCG) at the London School of Hygiene and Tropical Medicine, The Gambia London, UK<br>Erhart, Annette; MRC Unit The Gambia (MRCG) at the London School of Hygiene and Tropical Medicine, The Gambia<br>Diakite, Hamadoun ; University of Sciences Techniques and Technologies of Bamako, Malaria Research & Training Centre<br>Keita, Mohamed; University of Sciences Techniques and Technologies of Bamako Faculty of Medicine Odontostomatology<br>Piqueras, Mireia; Instituto de Salud Global Barcelona Barcelona, Catalunya, ES<br>González, Raquel; Instituto de Salud Global Barcelona Barcelona, Catalunya, ES<br>Menendez, Clara; Instituto de Salud Global Barcelona Barcelona, Catalunya, ES Barcelona, ES<br>Dorlo, Thomas; Netherlands Cancer Institute / Antoni van Leeuwenhoek Hospital, Amsterdam, the Netherlands; Department of Pharmacy, Uppsala University, Uppsala, Sweden<br>Sagara, Issaka; Universite des Sciences des Techniques et des Technologies de Bamako, Malaria Research and Training Center (MRTC)<br>Mens, Petra; Amsterdam University Medical Centres, Academic Medical Centre at the University of Amsterdam (AMC), Amsterdam, Netherlands

	Schallig, Henk; Amsterdam University Medical Centres, Academic Medical Centre at the University of Amsterdam (AMC), Amsterdam, Netherlands<br>D'Alessandro, Umberto; MRC Laboratories The Gambia<br>Kayentao, Kassoum; University of Sciences Techniques and Technologies of Bamako, Malaria Research & Training Centre
**Primary Subject Heading**:	Epidemiology
Secondary Subject Heading:	Infectious diseases
Keywords:	EPIDEMIOLOGY, Infection control < INFECTIOUS DISEASES, Tropical medicine < INFECTIOUS DISEASES, PARASITOLOGY

SCHOLARONE™
Manuscripts

Paper draft Version 12
August 15th, 2023

Administrative information

Efficacy and safety of pyronaridine-artesunate (PYRAMAX®) for the treatment of *P. falciparum* uncomplicated malaria in African pregnant women (PYRAPREG): study protocol for a phase 3, non-inferiority, randomized open-label clinical trial

Moussa Djimde[1,2¥], Japhet Kabalu Tshiongo[2,3¥], Hypolite Muhindo Mavoko[3], Halidou Tinto[4], Esperanca Sevene[5,6], Maminata Traoré[4], Anifa Vala[5], Salesio Macuacua[5], Bérenger Kaboré[4], Edgard Diniba Dabira[7], Annette Erhart[7], Hamadoun Diakité[1], Mohamed Keita[1], Mireia Piqueras[8], Raquel González[5,8], Clara Menéndez[5,8], Thomas P.C. Dorlo[9,10], Issaka Sagara[1], Petra F. Mens[2], Henk D. F. H. Schallig[2], Umberto D'Alessandro[7] and Kassoum Kayentao[1*]

[¥] These authors contributed equally

Author's Affiliations:

[1]Malaria Research and Training Center (MRTC), University of Sciences of Techniques and Technologies of Bamako (USTTB), Mali

[2]Amsterdam University Medical Centres, Academic Medical Centre at the University of Amsterdam (AMC), Amsterdam, Netherlands

[3]Department of Tropical Medicine, University of Kinshasa (UNIKIN), Kinshasa, Democratic Republic of the Congo

[4]Institut de Recherche en Sciences de la Santé (IRSS) – Unité de Recherche Clinique de Nanoro, Burkina Faso

[5]Centro de Investigação em Saúde da Manhiça (CISM), Mozambique

[6]Faculty of Medicine, Eduardo Mondlane University, Mozambique

[7]MRC Unit The Gambia (MRCG) at the London School of Hygiene and Tropical Medicine, The Gambia

[8]Barcelona Institute for Global Health (ISGlobal), Hospital Clínic-Universitat de Barcelona, Spain

[9] Netherlands Cancer Institute / Antoni van Leeuwenhoek Hospital, Amsterdam, the Netherlands

[10] Department of Pharmacy, Uppsala University, Uppsala, Sweden

For peer review only - http://bmjopen.bmj.com/site/about/guidelines.xhtml

Paper draft Version 12
August 15th, 2023

Trial registration:

PACTR202011812241529

Protocol version: 7.0 12 July 2022

Trial Sponsor and role

The trial sponsor is University of Sciences, Techniques, and Technologies of Bamako (USTTB). Hamdallaye ACI 2000, Rue 405 - Porte 359, Bamako

Téléphone : +223 20 29 04 07

http://usttb.edu.ml/

The Trial sponsor in the study has the responsibility to ensure that data and source documents granted for trial monitoring, audits, DSMB and Ethics Committee review and regulatory inspections as appropriate.

***Corresponding Author**: Kassoum Kayentao (kayentao@icermali.org)

Malaria Research and Training Center (MRTC), University of Sciences of Techniques and Technologies of Bamako (USTTB), Mali

For peer review only - http://bmjopen.bmj.com/site/about/guidelines.xhtml

Paper draft Version 12
August 15th, 2023

Abstract

Introduction: Malaria infection during pregnancy increases the risk of low birthweight and infant mortality and should be prevented and treated. Artemisinin-based combination treatments are generally well tolerated, safe and effective, the most used being artemether-lumefantrine (AL) and dihydroartemisinin-piperaquine (DP). Pyronaridine-artesunate (PA) is a new artemisinin-based combination. The main objective of this study is to determine the efficacy and safety of PA versus AL or DP when administered to pregnant women with confirmed *P. falciparum* infection in the second or third trimester. The primary hypothesis is the pair-wise non-inferiority of PA as compared to either AL or DP.

Methods and analysis: A phase 3, non-inferiority, randomized, open-label clinical trial to determine the safety and efficacy of AL, DP, and PA in pregnant women with malaria in 5 sub-Saharan, malaria-endemic countries (Burkina Faso, Democratic Republic of the Congo, Mali, Mozambique and The Gambia). A total of 1,875 pregnant women will be randomized to one of the treatment arms. Women will be actively monitored until the Day 63 post-treatment, at delivery and 4-6 weeks after delivery, and infants' health will be checked at their first birthday. The primary endpoint is the PCR-adjusted rate of adequate clinical and parasitological response at Day 42 in the per protocol population.

Ethics and Dissemination: This protocol has been approved by Ethics Committee for Health Research in Burkina Faso, the National Health Ethics Committee in Democratic Republic of Congo, the Ethics Committee of the Faculty of Medicine and Odontostomatology / Faculty of Pharmacy in Mali in Mali, The Gambia Government/MRCG Joint Ethics Committee and the National Bioethics Committee for Health in Mozambique. Written informed consent will be obtained from each individual prior to her participation in the study. The results will be published in peer reviewed Open Access Journals and presented at (inter)national conferences and meetings.

Keywords:

Malaria, pregnant women, pyronaridine-artesunate, artemether-lumefantrine, dihydroartemisinin-piperaquine, , Burkina Faso, Democratic Republic of the Congo, Mali, Mozambique, The Gambia.

For peer review only - http://bmjopen.bmj.com/site/about/guidelines.xhtml

Paper draft Version 12
August 15th, 2023

Strengths and limitations of this study

- This is randomized clinical trial that will provide evidence on the safety and efficacy of a newly registered Artemisinin-Based Combination pyronaridine-artesunate (PA) for the treatment of uncomplicated malaria in African pregnant women during the second and third trimester of pregnancy.
- The trial will generate additional data on the safety and efficacy of dihydroartemisinin-piperaquine, and artemether-lumefantrine and will increase substantially the treatment options for malaria in pregnancy.
- As PA is the last Artemisinin-Based Combination to have been registered for the treatment of malaria, the proposed trial will complement the information already available for malaria treatment in children and non-pregnant adults.
- Weaknesses include the lack of regular monitoring of children within the first year of birth and the non-inclusion of infected women in the first trimester.

For peer review only

For peer review only - http://bmjopen.bmj.com/site/about/guidelines.xhtml

Paper draft Version 12
August 15th, 2023

INTRODUCTION

Pregnancy is associated with hormonal changes and reduced immunity that make pregnant women more vulnerable to malaria [1]. Malaria during pregnancy increases the risk of low birth weight (LBW) which is associated with higher infant mortality.[2] The prevention and control of malaria during pregnancy is based on insecticide-treated nets (ITNs), intermittent preventive treatment during pregnancy (IPTp) with sulfadoxine-pyrimethamine (SP), and effective case management of clinical malaria, including anaemia.[3] The latter relies on artemisinin-based combination therapies (ACTs) which are well tolerated, safe and efficacious.[4] Their use is currently limited to the second and third trimester although the risk of miscarriage, stillbirths or major birth defects when used during the first trimester is better to quinine, the recommended drug.[5,6]

Currently available ACTs for the treatment of Plasmodium falciparum malaria during the second and third trimesters of pregnancy, namely artemether-lumefantrine (AL), artesunate-amodiaquine (ASAQ), mefloquine-artesunate (MAS) and dihydroartemisinin-piperaquine (DP), are generally safe and efficacious.[4] Nevertheless, when considering efficacy and tolerability, DP is probably the best option as it provides a long post-treatment prophylaxis and is well tolerated. DP is also considered for IPTp, as a possible alternative to SP where resistance to this treatment is high [7].[7] Its deployment as IPTp would significantly limit its use as curative treatment. There is the need for increasing the therapeutic options to treat malaria during the 2nd and 3rd trimesters of pregnancy. Pyronaridine-artesunate (PA) may be a possible candidate. PA tablets and granules (Pyramax®) have received a positive European Medicines Agency /Article 58 scientific opinion for the treatment of acute uncomplicated *P. falciparum* or *P. vivax* malaria.[8] The treatment (tablets) is currently registered in 19 African countries and 5 Asian countries. Pyramax (granules) is registered in 15 African countries.

To address this need, we designed a randomized open-label clinical trial to determine the efficacy, and safety of PA versus AL or DP when administered to pregnant women with confirmed *P. falciparum* infection in the second or third trimester.

The specific trial objectives are:

A. To compare the efficacy of PA versus AL or DP in terms of: (i) treatment success (see definition below) 28, 42 and 63 days after the start of treatment, with and without genotyping; (ii) parasite clearance time, including sub-microscopic malaria infections; (iii) gametocyte carriage and clearance; (iv) haematological recovery by Day 7, 14, 28,

For peer review only - http://bmjopen.bmj.com/site/about/guidelines.xhtml

42 and 63 after treatment and at delivery; (v) birth weight measured within 72 hours of delivery and (vi) placental *P. falciparum* malaria.

B. To describe the safety profile of PA, AL and DP in terms of: (i) tolerability and (ii) adverse events (AEs), including serious adverse events (SAEs) and adverse events of special interest (AESI), during the 63-day post-treatment follow-up (mother), at delivery (mother and baby), one month (mother and baby) and one year after the end of pregnancy (baby).

C. To explore the pharmacokinetics of pyronaridine in HIV-infected and non-HIV-infected pregnant women. This will be done on a small number of study subjects (60 women, 30 HIV uninfected and 30 HIV infected), in the 2 countries with the highest HIV prevalence, namely Democratic Republic of the Congo (DRC) and Mozambique.

METHODS AND ANALYSIS

Study setting

The trial will be implemented in five African countries with multiple sites of recruitment as indicated in table 1.

Table 1 Countries and Study site characteristics	
Burkina-Faso: Nanoro	The study site is situated in central-west of the country, 90 Km from Ouagadougou in the Nanoro health district catchment area. Malaria transmission is high and seasonal. *P. falciparum* is the most common species of malaria parasite. *Anopheles gambiae* is the main vector transmitting the disease.
Democratic Republic of the Congo: Lisungi	The trial will be carried out at Lisungi health centre, located in Kinshasa suburb, where malaria transmission is perennial. *P. falciparum* is the most common species of malaria parasite. *Anopheles funestus* and *Anopheles gambiae* are the main vector transmitting the disease.
Mali: San and Téné	San health centre is located at 440 km of northeast of Bamako. Téné is at about 50 km south of San. Malaria transmission is high and seasonal. *P. falciparum* is the most common species of malaria parasite. *Anopheles gambiae* is the main vector transmitting the disease.

For peer review only - http://bmjopen.bmj.com/site/about/guidelines.xhtml

Paper draft Version 12
August 15th, 2023

Mozambique: Manhiça District	Manhiça is located in southern Mozambique, 80 Km north of Maputo city. Malaria is endemic with perennial transmission. *P. falciparum* is the most common species of malaria parasite.
The Gambia: Basse, Demba Kunta Koto, Fatoto, Gambissara and Koina	The trial will be carried out in the Upper River Region (eastern part of the country), where the MRCG has a field station. There are 5 health facilities (Basse, Demba Kunta Koto, Fatoto, Gambissara and Koina) from which pregnant women can be recruited. Malaria transmission is seasonal. *P. falciparum* is the most common species of malaria parasite. *Anopheles gambiae* is the main vector transmitting the disease. *Anopheles arabiensis* and *Anopheles funestus* are the main vector transmitting the disease.

The different sites were chosen to include African pregnant women who are residents in different malaria transmission facies including the Gambia which is in pre-elimination phase. The study (recruitment) started in February 2021 and the planned end date is June 2024.

Patient and public involvement

Although patients were not involved in the design of the study, this project was designed to respond to their main concern which is the reduction of malaria adverse effect in pregnant population. The government of the five African countries were engaged through their National Malaria Control Programme which objective is to provide the evidence base to make an additional ACT available for malaria treatment in pregnancy. In case of a positive result, it will be important to integrate this new treatment in (inter) national guidelines. This will be obtained by disseminating as rapidly and efficiently as possible the trial's results to relevant stakeholders, e.g. WHO, National Malaria Control Programmes (NMCPs), and the scientific community working on malaria treatment. Through meetings in all the involved countries, permission from community, religious leaders, and women representatives has been sought before the trial start. Therefore, similar channels will be used to share the study results. Participation in our trial can be burdensome as participants may endure psychological, cost, and physical impacts. However, the project provided special attention such as closer supervision and treatment of any illness to enrolled patients. Also, transport cost has been provided to participants living far from the

For peer review only - http://bmjopen.bmj.com/site/about/guidelines.xhtml

recruitment centre. Nonetheless, it has been suggested to all participants to withdraw their consent any time if they fell burdened by the study procedures.

Study design

This is an open-label, multicentre, randomized, non-inferiority clinical trial comparing PA with AL and DP for the treatment of *P. falciparum* malaria in women in the second and third trimesters of pregnancy. The non-inferiority design was suggested based on the hypothesis that PA, which is a newly registered ACT is not worse than the other ACT in terms of safety (for AL) and efficacy (for DP). The rationale for 2 control groups is that AL is currently the most used treatment for malaria in pregnancy while DP seems to be the ACT with the best tolerability and efficacy profile.

The primary hypothesis is the pair-wise non-inferiority of PA as compared to either AL or DP, defined as a difference in the Day 42 PCR-adjusted adequate clinical and parasitological response (ACPR) of <5% (non-inferiority margin).

Participants and procedures

To be included, patients should satisfy all the inclusion criteria while none of the exclusion criteria should be present.

Inclusion criteria

1. Gestation ≥16 weeks and <37 weeks as assessed by ultrasound when possible. If not, height of the uterus or delay of menstruation will be used.
2. *P. falciparum* mono-infection (by microscopy) of any density, regardless of symptoms and HIV status.
3. Haemoglobin ≥7g/dL.
4. Age ≥15 years.
5. Residence within the health facility catchment area.
6. Willingness to adhere to study requirements and to deliver at the health facility.
7. Ability to provide written informed consent; if the woman is minor of age/not emancipated, the consent must be given by a parent or legal guardian according to national law (however, in this case, assent will be obtained to ensure that the woman herself is also freely willing to take part in the study).

For peer review only - http://bmjopen.bmj.com/site/about/guidelines.xhtml

8. For the PK study, HIV-infected women should be on first line anti-retroviral treatment for at least 6 months.

Exclusion criteria

1. Known allergy to the study drugs.
2. History of known pregnancy complications or poor obstetric history such as repeated stillbirths or eclampsia.
3. History or presence of major illnesses likely to influence pregnancy outcome.
4. Any significant illness at the time of screening requiring hospitalization, including:
   i. Severe malaria;
   ii. Any sign or symptom suggesting hepatic lesions (e.g. nausea with abdominal pain and icterus) or severe liver disease classified as B or C by the Child-Pugh score;
   iii. Known history or evidence of clinically significant cardiovascular disorders or family history of long QT syndrome.
5. Intent to move out of the study catchment area before delivery or delivery out of the catchment area.
6. Prior enrolment in the study.
7. Clear evidence of recent (1 week) treatment with antimicrobials with antimalarial activity (azithromycin, clindamycin, tetracycline, quinolones, cotrimoxazole and SP). For HIV-infected pregnant women to be included in the PK sub-study, cotrimoxazole use is not an exclusion criterion.
8. Twin/multiple pregnancy.
9. Known history of G6PD deficiency or sickle cell disease.

Randomisation

Sequence generation

Women recruited in the trial will be randomly allocated to one of the three treatment arms of the study according to a randomization list generated using R software prior any study activity. The block randomisation technique will be used to achieve balance in the allocation of participants to treatment arms. A varying size of the blocks will be setup to reduce selection bias by using random block sizes and keeping the investigator blind to the size of each block, particularly in this open randomized control arm study. The randomisation will be done per study site within each country and according to the specific sample size allocated to each site. The data management team in each country, in coordination with the study statistician and the

For peer review only - http://bmjopen.bmj.com/site/about/guidelines.xhtml

Paper draft Version 12
August 15th, 2023

study Data management Centre, will print the randomization code containing the study arm and put into a sealed envelope numbered sequentially and containing the treatment arm to which the patient should be allocated.

Recruitment

Women in the 2nd or 3rd trimester of pregnancy will be screened for malaria with a Rapid Diagnostic Test based on the detection of Histidine-Rich Protein 2 (HRP2) and Parasite lactate; positive results will be confirmed by microscopy. Although malaria slides will be read by two certified microscopists, there is a small change that mixed infections might be overlooked and this can be considered as a potential limitation of this study.

Women with a confirmed *P. falciparum* infection will be asked to provide a written informed consent covering all trial procedures and be assigned a screening number. All women meeting the entry criteria will be given a randomization number. Data of screened and randomized women will be kept in a logbook.

Investigational product and comparators

Pyronaridine-artesunate: Pyramax® (Shin Poong Pharmaceutical Company, Korea)

PA, is a film-coated tablet containing 180 mg pyronaridine tetraphosphate and 60 mg artesunate. The tablets should be taken orally once daily for three days and according to body weight as follows 24-<45Kg 2 tablets; 45-<65Kg 3 tablets; ≥65Kg 4 tablets. This dosage regimen provides a daily dose of 7.2-13.8 mg/kg pyronaridine and 2.4-4.6 mg/kg artesunate. PA can be administered at any time, regardless of food consumption.

Dihydroartemisinin-piperaquine (Alfasigma, Italy)

DP is a white film-coated tablet composed of 40 mg dihydroartemisinin and 320 mg piperaquine. DP tablets should be taken orally once daily for three days as follows 24-<36Kg 2 tablets; 36-<75Kg 3 tablets; ≥75Kg 4 tablets.[9],[10]

Artemether-lumefantrine: Coartem® (Novartis)

AL tablets contain 80 mg artemether and 480 mg lumefantrine. This is a fixed-dose combination of artemether (a semi synthetic artemisinin derivative) and lumefantrine (a slowly eliminated drug also referred to as benflumetol).[11] AL 80 mg/480 mg tablets should be taken orally twice daily for three days as follows to patients weighing 35 kg and above:

- 1st dose, at the time of initial diagnosis (Day 0): 1 tablet

For peer review only - http://bmjopen.bmj.com/site/about/guidelines.xhtml

- 2nd dose, at 8 hours after the 1st dose: 1 tablet
- 3rd dose, in the morning of the Day 1: 1 tablet
- 4th dose, in the evening of Day 1: 1 tablet
- 5th dose, in the morning of Day 2: 1 tablet
- 6th dose, in the evening of Day 2: 1 tablet

Investigator's brochures and all documentation on drug quality will be provided by the manufacturers. The drugs will be shipped to the different sites with temperature monitors recording the temperature continuously. Once at the sites, the drugs will be stored and used according to the manufacturers' instructions. Independent study monitors (CliniPharm) will ensure compliance with good clinical practice including good Investigational Product management practice.

Explanation for the choice of comparators.

Pregnant women (2nd or 3rd trimester) will be recruited for the study and allocated to one of the three treatment groups. The choice of two control arms (AL and DP) is justified by AL being the most commonly used treatment for malaria in African pregnant women, while DP has several advantages compared to AL, namely higher efficacy and longer post-treatment prophylaxis.

Study outcomes

Primary endpoint

The primary endpoint is treatment efficacy determined as the proportion of women with PCR-adjusted ACPR at Day 42, i.e. all women not having met the criteria of treatment failure (Supplementary Table 1). Recurrent infections classified by genotyping as new infection will not be considered as treatment failure.

Secondary endpoints

Safety is the main secondary endpoint and includes adverse events detected during active follow-up (63 days post-treatment), including significant changes in relevant laboratory values, those detected at delivery, at 4-6 weeks and 1 year after birth.

Others secondary endpoints include:

1. PCR-adjusted ACPR at Days 28 and 63;
2. PCR unadjusted ACPR on Days 28, 42 and 63;

For peer review only - http://bmjopen.bmj.com/site/about/guidelines.xhtml

3. Parasite and fever elimination time;
4. Gametocyte carriage and clearance;
5. Hematologic recovery, i.e., Hb changes between Day 0 and Days 7, 14, 28, 42, 63 and at delivery;
6. Placenta malaria (recent, past, and chronic infection);
7. Average BW and prevalence of low birth weight (<2,500g).

Exploratory endpoints

We will explore: (i) the drug exposure and key pharmacokinetic parameters of pyronaridine in HIV uninfected pregnant women and (ii) the drug exposure and key pharmacokinetic parameters of HIV-infected pregnant women on antiretroviral therapy. Drug exposure is defined as the area under the whole blood concentration versus time curve from zero to infinity, $AUC_{0-\infty}$. The main pharmacokinetic parameters that will be evaluated are absorption rate (k_a), drug clearance (CL/F) and volume of distribution (V_d/F).

Sample size

The sample size was estimated assuming an efficacy for PA (PCR adjusted at Day 42) of at least 95% and a non-inferiority margin of 5%; non-inferiority will be tested using raw pooling of country data, using a Wilson's interval of proportion difference. The lower limit of the Wilson score interval of 97.5% of the AL (or DP) proportion of ACPR - the PA proportion of ACPR must be greater than -5% to claim non-inferiority. In addition, an adjusted non-inferiority analysis will allow for country effect[12]. Assuming 20% of loss to follow-up, the total sample size for 90% power would be 3*500/0.8 = 1875, or 375 pregnant women per country and 125 per arm per country. The sample size calculated will maintain the power of the adjusted stratified non-inferiority test.

Study implementation and timeline

Participants' assignment into study arm is under the responsibility of study qualified physicians. All physician participating in PYRAPREG study have been trained in Good Clinical Practices and all study requirements.

Pregnant women fulfilling the inclusion/exclusion criteria will be recruited during antenatal clinics over a period of about 30 months. Scheduled visits will be at Day 3, 7 and then every week until Day 63 post-treatment. A window period is allowed if study subjects are unable to

For peer review only - http://bmjopen.bmj.com/site/about/guidelines.xhtml

attend on the scheduled date, i.e. ± 1-day for Day 7 and 14; ± 2 for Day 21 and 28; and ± 3 from Day 35 to Day 63. Women will be encouraged to attend the antenatal clinic between scheduled visits if sick.

Pregnant women recruited during the third trimester (before 37 weeks) may deliver before the end of the 63-day active follow up. In this case, the assessment at delivery will be done as planned but the active follow up will continue after delivery until Day 63. A blood sample to measure liver function test (LFT) and bilirubin will be taken within 48 hours of delivery from babies whose mothers delivered within 2 weeks of the inclusion in the trial.

The outcome of pregnancy, birth weight and maternal Hb will be collected as soon as possible after delivery. A placenta biopsy will be collected for later histopathological analysis. The new-born will be examined for congenital abnormalities. Both the mother and the new-born will be reassessed for any adverse event between 4 and 6 weeks and then after one year (only the baby). Patients will be assessed as summarized in the study visit schedule (Supplementary Table 2).

Data collection and management

Plans for assessment and collection of outcomes

Study visits: At each visit, both scheduled and unscheduled, the medical history since the last visit (including any treatment taken), current signs and symptoms (if any) will be collected. A blood sample for thick smear will be collected and the body temperature checked. Dried blood spots for later genotyping to distinguish between recrudescence and new infection will be collected at Day 0, before treatment, and at every study visit. Information on any adverse event will also be collected. Haematology (full blood count) will be performed at Days 0, 7, 14, 28, 42 and 63; biochemistry (Total and conjugated bilirubin, AST, ALT, alkaline phosphatase, and creatinine) at Day 0, 1, and 7. In the event of increased liver function tests (LFTs) >3xULN, the result will be verified by taking an additional sample to be analysed (within 24 hours). Subsequent blood samples for LFTs will be taken at 48-hour intervals until the results return to <2xULN. ECG will be performed at Day 0, before drug intake, and at Day 2 after drug intake. If abnormal at Day 2, ECG will be repeated at Day 7 and every week until return to normal. At the end of the active follow up, on Day 63, field assistants will visit the study subjects monthly to maintain the contact, but without collecting any data or biological samples.

Babies born from women who deliver within or 2 weeks after the active follow up will have a blood sample taken to measure LFT and bilirubin within 48 hours of delivery.

The outcome of pregnancy, birth weight and maternal Hb will be collected as soon as possible after delivery. A placenta biopsy will be collected, put immediately in 10% buffered formalin

For peer review only - http://bmjopen.bmj.com/site/about/guidelines.xhtml

container stored in 4°C at the study site for later histopathological analysis. The new-born will be examined for congenital abnormalities. Both the mother and the new-born will be reassessed twice after delivery for any adverse event: between 4 and 6 weeks and then after one year.

PCR analysis

Genotyping of recurrent infections will be performed by characterizing the merozoites surface protein 1 (*msp1*), *msp2* and glutamate rich protein (*glurp)* genes, single-copy genes of the *P. falciparum* genome. PCR amplification of deoxyribonucleic acid (DNA) from a single parasite clone will yield a unique amplification product. For all three genes, each PCR amplification product of different size is considered from a different *P. falciparum* clone and reflects a different genotype. For samples collected from the same patient on Day 0 and on the Day of recurrent parasitaemia (after Day 3), the length polymorphism of *msp1*, *msp2* and *glurp* will be determined, i.e., the number of bands in each PCR reaction and their respective sizes. The results will be interpreted as follows:

Recurrence: at least one polymorphism of identical length for each marker (*msp1*, *msp2* and *glurp*) is found in the sample collected on Day 0 and on the Day of the recurrent parasitaemia.

New infection: For at least one marker, the length polymorphism is different between the sample collected on Day 0 and on the Day of recurrent parasitaemia.

Indeterminate: Samples that did not give a result due to an inability to amplify DNA on Day 0 and/or on the Day of recurrent parasitaemia.

Analysis of placental samples

The placental biopsy samples will be processed and embedded in paraffin wax using standard .techniques. Paraffin sections 4 µm thick will be stained with haematoxylin-eosin. Placental biopsies after reading will be classified according to the following definitions[13]: (i) acute infection (parasite present, hemozoin absent or minimal deposition); (ii) chronic infection (parasites and heavy hemozoin deposition); (iii) past infection (no parasite but presence of hemozoin) or (iv) no infection (absence of parasites and hemozoin).

Haematological and biochemical analysis

Haematology (including haemoglobin) and biochemistry (including LFTs, i.e., AST, ALT, ALP, total and conjugated bilirubin) will be performed during active follow-up; haematology will be performed prior to the first dose of treatment, on Day 0, and then on Days 7, 14, 28, 42, and 63. An additional test will be performed at any unscheduled visit. In addition, only Hb will

For peer review only - http://bmjopen.bmj.com/site/about/guidelines.xhtml

be measured at delivery. Biochemistry will also be done on Day 0, prior to treatment, then on Day 1 and Day 7. If LFTs increase more than 3 times the upper limit normal (ULN), the result will be verified by taking another sample for analysis as soon as possible (within 24 hours of the initial sample). Subsequent blood samples for LFTs will be taken at 48-hour intervals until results return to < 2xULN.

Statistical methods

Statistical methods for primary and secondary outcomes

The baseline characteristics will be described by treatment group and site.

The primary analysis will be the assessment of the non-inferiority of the PA compared to the DP and AL for the PCR-adjusted ACPR at Day 42. It will use the combined data from the five countries, with adjustment for any centre effect, using an additive model for response rates (i.e., a generalised linear model with a Bernoulli error distribution and an identity link function). This will allow the evaluation of two pairwise treatment comparisons, i.e., PA versus AL, PA versus DP.

Efficacy analysis (primary end point)

For the efficacy analysis, both a m-ITT approach and a PP approach will be adopted, with PP analysis being the main approach, as recommended for equivalence studies. The m-ITT population will include all participants who have received any amount of study drug and have confirmed *P. falciparum* infection prior treatment.

The PP population will consist of all participants meeting the following predefined criteria:

1. Fulfilling the entry criteria specified in the clinical study protocol.
2. Completed treatment, including not having vomited the study drug or, if vomited, received a repeat dose that was not vomited.
3. No previous or concomitant medication that would interfere with treatment outcome.

The PP population will be identified after locking the database, just before the statistical analysis.

For the primary endpoint, i.e. treatment efficacy at Day 42, the proportion of participants with PCR-adjusted ACPR will be determined by treatment arm. Similar procedures will be applied to the m-ITT population.

For peer review only - http://bmjopen.bmj.com/site/about/guidelines.xhtml

Safety analysis (secondary end point)

The safety population will include all participants randomised and treated with at least one dose of the three antimalarial treatments. Standard safety report tables summarize and list safety data. All AEs will be coded using the most recent version of the Medical Dictionary for Regulatory activities (MedDRA) dictionary. Treatment emergent AEs will be defined as all AEs which started after the first administration of study drug. These AEs will be summarized by primary system organ class and preferred term, separately for each treatment regimen and overall. Similar summaries will be provided for treatment emergent AEs considered to be related to study treatments. In addition, treatment emergent AEs will be summarized by primary system organ class, preferred term, and maximal severity.

Vital signs and routine safety laboratory data will be summarised descriptively by treatment regimen and overall, by time point. Absolute values and changes from baseline will be presented. Safety laboratory data will be classified according to the normal ranges (below, within, above) and summaries of changes from baseline in these categories will be provided by treatment regimen and overall. Further, safety laboratory values will be classified according to Common Terminology Criteria for Adverse Events (CTCAE) and shift tables of the baseline CTCAE category versus post baseline categories will be presented.

Pharmacokinetic analysis

Pyronaridine exposure in patients' blood will be assessed in pregnant women, both infected and non-infected by HIV, by determining pyronaridine concentrations in EDTA whole blood samples collected on day 0 before and 1 hour (hr), 2hr, 6hr and 10hr after the first PA dose, onday 1 (24hr after the first PA dose while before the second PA dose), day 2 (before the last PA dose), and subsequently on day 7, 14, 21, 28 and 42 after initiation of treatment. A population pharmacokinetic approach using nonlinear mixed effects modelling will be employed to analyse the pharmacokinetic data. A compartmental population PK model will be developed, including a stochastic model describing between-subject and residual variability. In short, the main parameter of interest is overall whole blood pyronaridine exposure, defined as the area under the concentration-time curve until infinity ($AUC_{0\text{-inf}}$), which will be characterized based on the final individual model parameter estimates and compared to a separate cohort of non-pregnant malaria patients treated with PA. The effect of pregnancy will be evaluated and quantified either as a binary or continuous (estimated gestational age) covariate on all primary estimated pharmacokinetic parameters (such as k_a, CL/F and V_d/F). Furthermore, we will explore the effect of HIV-background (disease effects and potential

For peer review only - http://bmjopen.bmj.com/site/about/guidelines.xhtml

antiretroviral drug-drug interactions) on the pharmacokinetics of pyronaridine by evaluating HIV status as covariate. Statistical significance and selection of covariates in nested population PK models will be based on a likelihood ratio test to evaluate the difference in model fit.

Study monitoring

The trial will be evaluated by monitors (a Clinical Research Organization will be contracted) (pre-study visit) for its preparedness to carry out, following by regular monitoring and closeout visits.

The coordination of the whole project is in the hand of the sponsor, which will be the primary contact. He will be assisted by appropriate administrative and financial staff. A Coordinating Committee (CC) including one member of each institution will be main decision body of the consortium. For the trial, there will be three entities involved in its implementation and management, namely the Data Safety and Monitoring Board (DSMB), the Trial Steering Committee (TSC), and the Trial Management Group (TMG). Both DSMB and TSC are composed of independent experts to provide the overall supervision of the trial, monitor trial progress and advise on scientific credibility.

Ethics and dissemination

This protocol has been approved by Ethics Committee for Health Research (CERS) in Burkina Faso (Reference: 2020-3-047), the National Health Ethics Committee (CNES) in Democratic Republic of Congo (Reference: 169/CNES/BN/PMMF/2019), the Ethics Committee of the Faculty of Medicine and Odontostomatology (FMOS) / Faculty of Pharmacy (FPHA) in Mali (Reference: 2020/46/CE/FMOS/FAPH) in Mali, The Gambia Government/MRCG Joint Ethics Committee (Reference: 21818) and the National Bioethics Committee for Health (CNBS) in Mozambique (Reference: 313/CNBS/20).

Written informed consent will be taken from each individual prior to her participation in the study, the study investigators.

The outcomes of the project will be communicated to the National Malaria Control Programme of the respective countries and to the Global Malaria Programme of the World Health Organization. The results will be published in peer reviewed Open Access Journals and presented at (inter)national conferences and meetings.

For peer review only - http://bmjopen.bmj.com/site/about/guidelines.xhtml

Paper draft Version 12
August 15th, 2023

A work package is focused on the development of a plan for internal and external project communication, on the development of communication tools, and on the dissemination and exploitation of the project's findings. All these activities will be in line with H2020 guidelines on dissemination and publication of results and will highlight the contribution of the EDCTP in tackling societal and health challenges.

Trial status

Active recruitment

Abbreviations

ACPR	Adequate clinical and parasitological response
ACT	Artemisinin-based combination therapy
AE	Adverse event
AESI	Adverse event of particular interest
AL	Artemether-lumefantrine
ALP	Alkaline phosphatase
ALT	Alanine aminotransferase
AMC	Academic Medical Centre
AST	Aspartate aminotransferase
CC	Coordinating Committee
CISM	Centro de Investigação em Saúde da Manhiça
CRO	Contract Research Organization
CRUN	Clinical Research Unit of Nanoro
CTCAE	Common Terminology Criteria for Adverse Events
DNA	deoxyribonucleic acid
DP	Dihydroartemisinin-piperaquine
DSMB	Data Safety Monitoring Board
eCRF	Electronic case report form
EDCTP	European & Developing Countries Clinical Trials Partnership
ETF	Early treatment failure
FAPH	Faculty of Pharmacy
FMOS	Faculty of Medicine and Odontostomatology

For peer review only

For peer review only - http://bmjopen.bmj.com/site/about/guidelines.xhtml

GCP	Good Clinical Practice
glurp	Glutamate rich protein
HIV	Human immunodeficiency virus
HS-RDT	Highly sensitive rapid diagnostic test
IPTp-SP	Intermittent preventive treatment during pregnancy with sulfadoxine-pyrimethamine
ITN	Insecticide Treated Net
IRSS	Institut de Recherche en Sciences de la Santé
MedDRA	Medical Dictionary for Regulatory activities
mITT	Modified Intention-to-treat
LFT	Liver Function Tests
LBW	Low birth weight
LCF	Late Clinical Failure
LPF	Late parasitological failure
LTF	Late treatment failure
MAS	Mefloquine-artesunate
MMV	Medicine of Malaria Venture
MRTC	Malaria Research and Training Center
MRCG	The MRC Unit The Gambia
msp1	Merozoites Surface Protein gene 1
NKI	Nederlands Kanker Institutu
NOAEL	No observed adverse effect level
P. falciparum	*Plasmodium falciparum*
P. vivax	*Plasmodium vivax*
PA	Pyronaridine-artesunate
PCR	Polymerase chain reaction
PP	Per protocol analysis
REDCap	Research Electronic Data Capture
SAE	Serious adverse event
SP	Sulfadoxine-pyrimethamine
TMG	Trial management group
TSC	Trial Steering Committee
ULN	Upper Limit Normal

For peer review only

For peer review only - http://bmjopen.bmj.com/site/about/guidelines.xhtml

Paper draft Version 12
August 15th, 2023

UNIKIN	University of Kinshasa
USTTB	University of Sciences of Techniques and Technologies of Bamako
WANECAM	West African Network for Antimalarial Drugs
WHO	World Health Organization

Acknowledgments

The consortium thanks Shinpoong Pharm.co., LTd for providing the study drug (pyronaridine-artesunate: Pyramax) and the Medicine of Malaria Venture (MMV) for technical support and assistance to this ongoing clinical trial. The project would like to thank patients and their advisors for their contribution to the study implementation.

Authors' information

Moussa Djimde: mdjimde@icermali.org

Japhet Kabalu Tshiongo: kabalujaphet@gmail.com

Hypolite Muhindo-Mavoko: hypomavoko@gmail.com

Halidou Tinto: halidoutinto@gmail.com

Esperanca Sevene: esevene68@gmail.com

Maminata Traoré: traoré_maminata@yahoo.fr

Anifa Vala: anifa.vala@manhica.net

Salesio Macuacua: salesio.macuacua@manhica.net

Bérenger Kaboré: kaboreberenger@gmail.com

Edgard Diniba Dabira: edabira@mrc.gm

Annette Erhart: Annette.Erhart@lshtm.ac.uk

Hamadoun Diakite: diakiteh@icermali.org

Mohamed Keita: medkeita@gmail.com

Mireia Piqueras: mireia.piqueras@isglobal.org

Raquel González: Raquel.gonzalez@isglobal.org

Clara Menéndez: Clara.menendez@isglobal.org

Thomas P. C. Dorlo: thomas.dorlo@farmaci.uu.se Issaka Sagara: isagara@icermali.org

Petra Mens: p.f.mens@amsterdamumc.nl

Henk Schallig: h.d.schallig@amsterdamumc.nl

Umberto D'Alessandro: udalessandro@mrc.gm

Kassoum Kayentao: kayentao@icermali.org

For peer review only - http://bmjopen.bmj.com/site/about/guidelines.xhtml

Paper draft Version 12
August 15th, 2023

Author's contributions

The following co-authors were responsible for the conception and design of the clinical trial : KK-Kassoum Kayentao, UDA-Umberto D'Alessandro, HMM-Hypolite Muhindo-Mavoko, HT-Halidou Tinto, MT-Maminata Traore, ES-Esperanca Sevene, MP-Mireia Piqueras, RG-Raquel Gonzalez, CM-Clara Menendez, TPCD-Thomas P.C. Dorlo, PFM-Petra F. Mens, HDFHS-Henk D. Schallig. The first draft of the paper was written by MD-Moussa Djimde and JKT-Japhet Kabalu Tshiongo. IS-Issaka Sagara is responsible for the statistical analysis. All authors and the following (AV-Anifa Vala, SM-Salesio Macuacua, BK-Berenger Kabore, EDD-Edgard Diniba Dabira, AE-Annette Erhart, HD-Hamadoun Diakite, MK-Mohamed Keita) contributed to critical review and approved the final manuscript.

Competing interests

None of the authors declare a conflict of interests.

Funding statement

This project is part of the EDCTP2 programme supported by the European Union (grant number RIA2017MC-2025-PYRAPREG).

REFRENCES

1 Takem EN, D'Alessandro U. MALARIA IN PREGNANCY. *Mediterr J Hematol Infect Dis* 2013;**5**:e2013010. doi:10.4084/mjhid.2013.010

2 Desai M, ter Kuile FO, Nosten F, *et al.* Epidemiology and burden of malaria in pregnancy. Lancet Infectious Diseases. 2007. doi:10.1016/S1473-3099(07)70021-X

3 Rogerson SJ, Desai M, Mayor A, *et al.* Burden, pathology, and costs of malaria in pregnancy: new developments for an old problem. The Lancet Infectious Diseases. 2018. doi:10.1016/S1473-3099(18)30066-5

4 D'Alessandro U, Hill J, Tarning J, *et al.* Treatment of uncomplicated and severe malaria during pregnancy. The Lancet Infectious Diseases. 2018. doi:10.1016/S1473-3099(18)30065-3

5 Dellicour S, Sevene E, McGready R, *et al.* First-trimester artemisinin derivatives and quinine treatments and the risk of adverse pregnancy outcomes in Africa and Asia: A meta-analysis of observational studies. *PLoS Medicine* Published Online First: 2017. doi:10.1371/journal.pmed.1002290

For peer review only - http://bmjopen.bmj.com/site/about/guidelines.xhtml

6 Marrelli MT, Brotto M, Sharma H, *et al.* The effect of malaria and anti-malarial drugs on skeletal and cardiac muscles. *Malaria Journal* 2016;**15**:524. doi:10.1186/s12936-016-1577-y

7 Meghna Desai, Jenny Hill, Silke Fernandes, Patrick Walker, Christopher Pell, Julie Gutman, Kassoum Kayentao, Raquel Gonzalez, Jayne Webster, Brian Greenwood, Michel Cot FOTK. Prevention of malaria in pregnancy. *Lancet Infect Dis* 2018;**18(4):e119**.

8 European Public Assessment Report (EPAR). Assessment report; Pyramax. https://www.ema.europa.eu/en/documents/medicine-outside-eu/pyramax-public-assessment-report_en.pdf; Accessed on 17 February 2021. 2016.

9 D'Alessandro U. Existing antimalarial agents and malaria-treatment strategies. Expert Opinion on Pharmacotherapy. 2009. doi:10.1517/14656560902942319

10 Artemisinin-Based Treatments in Pregnant Women with Malaria. *New England Journal of Medicine* Published Online First: 2016. doi:10.1056/nejmc1604709

11 Muhindo Mavoko H, Nabasumba C, Tinto H, *et al.* Impact of retreatment with an artemisinin-based combination on malaria incidence and its potential selection of resistant strains: study protocol for a randomized controlled clinical trial. *Trials 2013 14:1* 2013;**14**:1–15. doi:10.1186/1745-6215-14-307

12 Yan X, Su XG. Stratified Wilson and Newcombe Confidence Intervals for Multiple Binomial Proportions. *Statistics in Biopharmaceutical Research* Published Online First: 2010. doi:10.1198/sbr.2009.0049

13 Ismail MR, Ordi J, Menendez C, *et al.* Placental pathology in malaria: A histological, immunohistochemical, and quantitative study. *Human Pathology* Published Online First: 2000. doi:10.1016/S0046-8177(00)80203-8

For peer review only

For peer review only - http://bmjopen.bmj.com/site/about/guidelines.xhtml

Table legend

Table 1: Definition of early and late treatment failures

Table 2: Study visit schedule

Table 1: Definition of early and late treatment failures

**Early treatment failure (ETF)** one of the following	**Late treatment failure (LTF)**	
1) Development of danger signs or severe malaria on Days 0-3 with parasitaemia<br><br>2) Presence of parasitaemia on Day 3 with fever (axillary temperature ≥ 37.5°C)		Late Clinical Failure (LCF):<br>1) Development of danger signs or severe malaria on any day after Day 3 in the presence of parasitaemia, without having previously met any of the ETF criteria;<br>2) Presence of parasitaemia and fever on any day after Day 3, without having previously met the ETF criteria.<br><br>Late parasitological failure (LPF):<br>Presence of parasitaemia on any day, starting on Day 7, and axillary temperature <37.5 ºC, without previously meeting any of the ETF or LCF criteria.

For peer review only

For peer review only

Table 2: Study visit schedule

**Day**	**0**	**1**	**2**	**3**	**7**(± 1)	**14** (± 1)	**21** (± 2)	**28** (± 2)	**35** (± 3)	**42** (± 3)	**49-56** (± 3)	**63** (± 3)	**Any other day**[1]	**Delivery**	**4-6 weeks post-end of pregnancy**	**1-year post-end of pregnancy**
History (symptoms)	X	X	X	X	X	X	X	X	X	X	X	X	X			
Informed consent	X															
Examination (clinical)	X	X	X	X	X	X	X	X	X	X	X	X	X	X		
Vital signs	X	X	X	X	X	X	X	X	X	X	X	X	X	X		
Foetal viability	X			X	X	X	X	X	X	X	X	X	X			
Blood Pressure	X	X	X	X	X	X	X	X	X	X	X	X	X	X		
Body Temperature	X	X	X	X	X	X	X	X	X	X	X	X	X	X		
ECG	X		X		X[2]											
Blood slide[3]	X	X	X	X	X	X	X	X	X	X	X	X	X	X		
Filter paper for genotyping	X	X	X	X	X	X	X	X	X	X	X	X	X	X		
Adverse drug reactions	X	X	X	X	X	X	X	X	X	X	X	X	X	X	X	
Concomitant medications	X	X	X	X	X	X	X	X	X	X	X	X	X	X	X	
Haematology	X				X	X		X		X		X	X	X[6]		
Treatment administration	X	X	X													
Blood sample for PK[4]	X	X	X		X	X	X	X		X						
Biochemistry[5]	X	X			X											
Urine analysis	X							X		X		X				
Placenta biopsy														X		
Newborn Assessment														X[8]	X	
Newborn LFT														X[7]		
Infant assessment																X

[1]Spontaneous attendance to the health facility (unscheduled visit); [2]ECG at Day 7 if abnormal at Day 2; [3]Thick and thin blood film; [4]Four blood samples at scheduled times, first sampling before treatment administration;[5] In the event of increased LFTs >3xULN, the result will be verified by taking a further sample for analysis as soon as possible (within 24 hours of the original sample). Subsequent blood samples for LFTs will be taken at 48-hour intervals until the results return to ≤2xULN;[6] Only Hb; [7]If delivery within or 2 weeks after the active follow up; [8]Blood sample collected systematically for dried blood spots, blood slide and Hb.

For peer review only

STANDARD PROTOCOL ITEMS: RECOMMENDATIONS FOR INTERVENTIONAL TRIALS

SPIRIT 2013 Checklist: Recommended items to address in a clinical trial protocol and related documents*

Section/item	Item No	Description	Addressed on page number
**Administrative information**			
Title	1	Descriptive title identifying the study design, population, interventions, and, if applicable, trial acronym	________1______
Trial registration	2a	Trial identifier and registry name. If not yet registered, name of intended registry	________2______
	2b	All items from the World Health Organization Trial Registration Data Set	______________
Protocol version	3	Date and version identifier	_______2________
Funding	4	Sources and types of financial, material, and other support	______ 18-19______
Roles and responsibilities	5a	Names, affiliations, and roles of protocol contributors	_____18________
	5b	Name and contact information for the trial sponsor	_____2_________
	5c	Role of study sponsor and funders, if any, in study design; collection, management, analysis, and interpretation of data; writing of the report; and the decision to submit the report for publication, including whether they will have ultimate authority over any of these activities	_____2_________
	5d	Composition, roles, and responsibilities of the coordinating centre, steering committee, endpoint adjudication committee, data management team, and other individuals or groups overseeing the trial, if applicable (see Item 21a for data monitoring committee)	_____2_________

For peer review only - http://bmjopen.bmj.com/site/about/guidelines.xhtml

Introduction

Background and rationale	6a	Description of research question and justification for undertaking the trial, including summary of relevant studies (published and unpublished) examining benefits and harms for each intervention	__5_________
	6b	Explanation for choice of comparators	___5, 9-10______
Objectives	7	Specific objectives or hypotheses	____5,6_______
Trial design	8	Description of trial design including type of trial (eg, parallel group, crossover, factorial, single group), allocation ratio, and framework (eg, superiority, equivalence, noninferiority, exploratory)	_____5,7_______

Methods: Participants, interventions, and outcomes

Study setting	9	Description of study settings (eg, community clinic, academic hospital) and list of countries where data will be collected. Reference to where list of study sites can be obtained	_____8________
Eligibility criteria	10	Inclusion and exclusion criteria for participants. If applicable, eligibility criteria for study centres and individuals who will perform the interventions (eg, surgeons, psychotherapists)	____8-9________
Interventions	11a	Interventions for each group with sufficient detail to allow replication, including how and when they will be administered	__9-10________
	11b	Criteria for discontinuing or modifying allocated interventions for a given trial participant (eg, drug dose change in response to harms, participant request, or improving/worsening disease)	____9-10_______
	11c	Strategies to improve adherence to intervention protocols, and any procedures for monitoring adherence (eg, drug tablet return, laboratory tests)	____8-9,12-13___
	11d	Relevant concomitant care and interventions that are permitted or prohibited during the trial	____9_________
Outcomes	12	Primary, secondary, and other outcomes, including the specific measurement variable (eg, systolic blood pressure), analysis metric (eg, change from baseline, final value, time to event), method of aggregation (eg, median, proportion), and time point for each outcome. Explanation of the clinical relevance of chosen efficacy and harm outcomes is strongly recommended	__10-11_______
Participant timeline	13	Time schedule of enrolment, interventions (including any run-ins and washouts), assessments, and visits for participants. A schematic diagram is highly recommended (see Figure)	_____12, 25__

For peer review only - http://bmjopen.bmj.com/site/about/guidelines.xhtml

Sample size	14	Estimated number of participants needed to achieve study objectives and how it was determined, including clinical and statistical assumptions supporting any sample size calculations	_11____________
Recruitment	15	Strategies for achieving adequate participant enrolment to reach target sample size	__9, 12________

Methods: Assignment of interventions (for controlled trials)

Allocation:

Sequence generation	16a	Method of generating the allocation sequence (eg, computer-generated random numbers), and list of any factors for stratification. To reduce predictability of a random sequence, details of any planned restriction (eg, blocking) should be provided in a separate document that is unavailable to those who enrol participants or assign interventions	___9__________
Allocation concealment mechanism	16b	Mechanism of implementing the allocation sequence (eg, central telephone; sequentially numbered, opaque, sealed envelopes), describing any steps to conceal the sequence until interventions are assigned	______9_______
Implementation	16c	Who will generate the allocation sequence, who will enrol participants, and who will assign participants to interventions	_______9______
Blinding (masking)	17a	Who will be blinded after assignment to interventions (eg, trial participants, care providers, outcome assessors, data analysts), and how	________9_____
	17b	If blinded, circumstances under which unblinding is permissible, and procedure for revealing a participant's allocated intervention during the trial	______8_______

Methods: Data collection, management, and analysis

Data collection methods	18a	Plans for assessment and collection of outcome, baseline, and other trial data, including any related processes to promote data quality (eg, duplicate measurements, training of assessors) and a description of study instruments (eg, questionnaires, laboratory tests) along with their reliability and validity, if known. Reference to where data collection forms can be found, if not in the protocol	_____12-13_____
	18b	Plans to promote participant retention and complete follow-up, including list of any outcome data to be collected for participants who discontinue or deviate from intervention protocols	______12-13___

For peer review only - http://bmjopen.bmj.com/site/about/guidelines.xhtml

Data management	19	Plans for data entry, coding, security, and storage, including any related processes to promote data quality (eg, double data entry; range checks for data values). Reference to where details of data management procedures can be found, if not in the protocol	_______12-13____
Statistical methods	20a	Statistical methods for analysing primary and secondary outcomes. Reference to where other details of the statistical analysis plan can be found, if not in the protocol	_____14-15_______
	20b	Methods for any additional analyses (eg, subgroup and adjusted analyses)	___14-15________
	20c	Definition of analysis population relating to protocol non-adherence (eg, as randomised analysis), and any statistical methods to handle missing data (eg, multiple imputation)	_____15__________
**Methods: Monitoring**			
Data monitoring	21a	Composition of data monitoring committee (DMC); summary of its role and reporting structure; statement of whether it is independent from the sponsor and competing interests; and reference to where further details about its charter can be found, if not in the protocol. Alternatively, an explanation of why a DMC is not needed	_____16__________
	21b	Description of any interim analyses and stopping guidelines, including who will have access to these interim results and make the final decision to terminate the trial	_____14_____
Harms	22	Plans for collecting, assessing, reporting, and managing solicited and spontaneously reported adverse events and other unintended effects of trial interventions or trial conduct	___14_______
Auditing	23	Frequency and procedures for auditing trial conduct, if any, and whether the process will be independent from investigators and the sponsor	_____16__________
**Ethics and dissemination**			
Research ethics approval	24	Plans for seeking research ethics committee/institutional review board (REC/IRB) approval	______16_________
Protocol amendments	25	Plans for communicating important protocol modifications (eg, changes to eligibility criteria, outcomes, analyses) to relevant parties (eg, investigators, REC/IRBs, trial participants, trial registries, journals, regulators)	__16____________

Consent or assent	26a	Who will obtain informed consent or assent from potential trial participants or authorised surrogates, and how (see Item 32)	___16__________
	26b	Additional consent provisions for collection and use of participant data and biological specimens in ancillary studies, if applicable	____NA_________
Confidentiality	27	How personal information about potential and enrolled participants will be collected, shared, and maintained in order to protect confidentiality before, during, and after the trial	____16_________
Declaration of interests	28	Financial and other competing interests for principal investigators for the overall trial and each study site	_____20_________
Access to data	29	Statement of who will have access to the final trial dataset, and disclosure of contractual agreements that limit such access for investigators	___9.13. 17____
Ancillary and post-trial care	30	Provisions, if any, for ancillary and post-trial care, and for compensation to those who suffer harm from trial participation	_______NA____
Dissemination policy	31a	Plans for investigators and sponsor to communicate trial results to participants, healthcare professionals, the public, and other relevant groups (eg, via publication, reporting in results databases, or other data sharing arrangements), including any publication restrictions	___16__________
	31b	Authorship eligibility guidelines and any intended use of professional writers	_____None_____
	31c	Plans, if any, for granting public access to the full protocol, participant-level dataset, and statistical code	_____None_____
**Appendices**			
Informed consent materials	32	Model consent form and other related documentation given to participants and authorised surrogates	_____26-40____
Biological specimens	33	Plans for collection, laboratory evaluation, and storage of biological specimens for genetic or molecular analysis in the current trial and for future use in ancillary studies, if applicable	_____12-14____

*It is strongly recommended that this checklist be read in conjunction with the SPIRIT 2013 Explanation & Elaboration for important clarification on the items. Amendments to the protocol should be tracked and dated. The SPIRIT checklist is copyrighted by the SPIRIT Group under the Creative Commons "Attribution-NonCommercial-NoDerivs 3.0 Unported" license.

For peer review only - http://bmjopen.bmj.com/site/about/guidelines.xhtml
